# Amorphous Engineering Driving d‐Orbital High Spin Configuration for Almost 100% ^1^O_2_‐Mediated Fenton‐Like Reactions

**DOI:** 10.1002/advs.202503665

**Published:** 2025-04-26

**Authors:** Juanjuan Qi, Qian Bai, Xiuhui Bai, Hongfei Gu, Siyue Lu, Siyang Chen, Qiangwei Li, Xudong Yang, Jianhui Wang, Lidong Wang

**Affiliations:** ^1^ MOE Key Laboratory of Resources and Environmental Systems Optimization College of Environmental Science and Engineering North China Electric Power University Beijing 102206 P. R. China; ^2^ School of Chemistry Beijing Advanced Innovation Center for Biomedical Engineering Key Laboratory of Bio‐Inspired Smart Interfacial Science and Technology Beihang University Beijing 100191 P. R. China; ^3^ Institute of Energy Resources Hebei Academy of Sciences Shijiazhuang 050081 P. R. China

**Keywords:** almost 100% ^1^O_2_, amorphous engineering, high spin state, ROS transformation, single atom catalysts

## Abstract

The inherent atomic disorder in amorphous materials leads to unsaturated atomic sites or dangling bonds, effectively modulating the material's electronic states and rendering it an ideal platform for the growth of single atoms. Herein, the electronic structure of isolated cobalt atoms anchored on amorphous carbon nitride (Co‐ACN) is modulated through a substrate amorphization engineering, enabling the thorough removal of pazufloxacin (PZF) in 1 min with a high reaction rate constant (*k*_1_) of 3.504 min^−1^ by peroxymonosulfate (PMS) activation. Experiments and theoretical calculations reveal that Co‐ACN exhibited a higher coordination environment (Co‐N_3_) compared to crystalline Co‐CCN (Co‐N_2_). Meanwhile, the t_2g_ energy level enhancement of Co 3d orbital promotes electron transition from t_2g_ to e_g_, inducing more unpaired electrons and thereby driving the transition from a low‐spin state (LS, t_2g_^6^e_g_^1^) to a high‐spin state (HS, t_2g_^5^e_g_^2^). The HS Co‐ACN optimized the d‐band center, boosted the electronic transfer, and weakened the interaction between Co 3d and O 2p orbitals of HSO_5_^−^, thereby enabling nearly 100% selective singlet oxygen (^1^O_2_) generation, whereas Co‐CCN yielded coexisting reactive oxygen species (ROS). This work opens up a new paradigm for regulating the electronic structure of single‐atom catalysts at the atomic scale.

## Graphical abstract

Through an amorphization strategy, the electronic structure of single‐atom Co is effectively modulated from a low spin state (Co‐CCN) to a high spin state (Co‐ACN). The rearrangement of the electronic configuration optimizes the d‐band center resulting in the selectivity of ^1^O_2_ with almost 100%. The Co‐ACN/PMS system demonstrates robust resistance to interference and high stability.

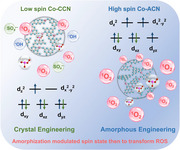

## 1 Introduction

Heterogeneous PMS‐activated Fenton‐like reactions have been established as a promising strategy for antibiotic degradation in wastewater^[^ ^1^ ^]^ due to the generation of highly reactive oxygen species through interactions between persulfates and active sites.^[^ ^2^ ^]^ Notably, ^1^O_2_, produced via non‐radical pathways, exhibits superior environmental stability and degradation selectivity compared to hydroxyl radicals (^•^OH) and sulfate radicals (SO_4_^•−^), owing to its extended lifetime, broad pH tolerance, and enhanced selectivity.^[^ ^3^ ^]^ However, the selective generation of ^1^O_2_ remains a significant challenge, as it is frequently accompanied by the formation of competitive by‐products such as SO_4_^•−^ and ^•^OH. This underscores the need for precise atomic‐level manipulation of catalysts to achieve selective ^1^O_2_ generation.^[^ ^4^ ^]^

To date, N‐doped carbon‐supported single‐atom catalysts (SACs) have shown considerable promise for improving selectivity and efficiency in antibiotic degradation through nonradical‐dominated pathways.^[^ ^5^ ^]^ Zhan et al. demonstrated that optimizing the coordination environment enhanced the adsorption energy of PMS at CoN_2+2_ sites compared with CoN_4_ sites, and then improved the yield of ^1^O_2_.^[^ ^6^ ^]^ Besides, amino modification of Co‐SACs reduced the gap between the d‐band center and the Fermi level, promoted charge transfer, and lowered the free energy barrier for PMS activation.^[^ ^7^ ^]^ Therefore, the carbon substrates employed to anchor atomic Co play a crucial role in influencing electronic metal‐support interactions.^[^ ^8^ ^]^ Amorphous nanomaterials are rich in intrinsic defects and coordination‐unsaturated sites, which can effectively anchor single metal atoms, preventing the aggregation of metal atoms into clusters and making it an ideal incubator for single‐atom growth.^[^ ^9^ ^]^ The metal‐support interactions induced by single atoms on amorphous and crystalline carriers exhibit significant differences, resulting in a distinct structure‐activity relationship in the catalytic process. Significantly, heat treatment can disrupt the intraplanar hydrogen bonding and weak interlayer van der Waals forces in crystalline graphitic carbon nitride (CCN), thereby achieving homogeneous amorphization, making it a unique class of support for SACs.^[^ ^10^ ^]^ Guo et al.^[^ ^11^ ^]^ reported that the disordered atomic structure in amorphous graphitic carbon nitride (ACN) results in a higher number of dangling bonds and unsaturated coordination sites, making ACN more effective than crystalline CCN for trapping single metal atoms.

In recent years, various active drugs have been detected in water bodies, posing a threat to both the ecological environment and human health.^[^ ^12^ ^]^ PZF, a representative antibiotic, was frequently detected in pharmaceutical wastewater, which was considered a target contaminant for the degradation.^[^ ^13^ ^]^ Herein, we significantly improved the catalytic performance of the Fenton‐like reaction based on PMS by tuning the electronic structure of single‐atom Co catalysts through an amorphization strategy. Compared to Co‐CCN (Co‐N_2_, low spin state) in crystal engineering, Co‐ACN in amorphous engineering features a unique coordination environment (Co‐N_3_) and a high spin state, enabling PZF degradation by generating almost 100% ^1^O_2_. Furthermore, the narrowed gap between the e_g_^*^ and t_2g_^*^ orbitals upon PMS adsorption on Co‐ACN enhances electron transfer, increasing SO_5_^•−^ generation, while the weakened PMS‐Co bonding due to the high spin Co state enables ≈100% selective production of ¹O_2_. Meanwhile, Co‐ACN exhibits optimal Gibbs free energies in each step, which is crucial for its excellent catalytic performance. Benefiting from the 100% active ¹O_2_species, Co‐ACN demonstrates excellent degradation performance for a wide range of organic pollutants across a broad pH range, along with good cycling stability.

## 2 Results and Discussion

### 2.1 Characterizations of the Catalysts

**Figure 1:**
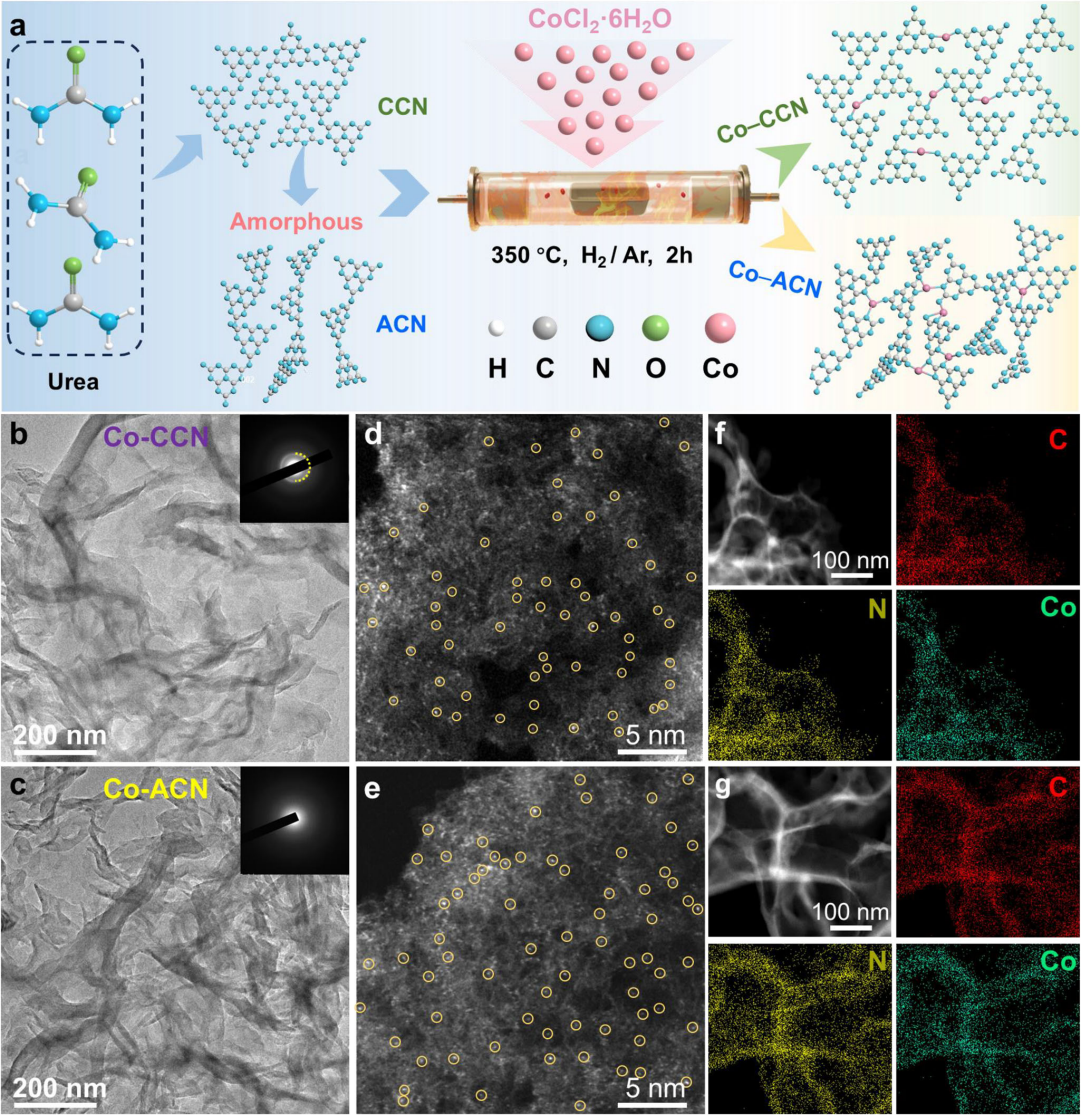
Structural characterization of Co‐ACN and Co‐CCN. a) Schematic illustration of the formation of Co‐ACN and Co‐CCN. C, N, O, H, and Co atoms are represented by grey, sky blue, green, white, and pink balls, respectively, b, c) Transmission electron microscopy (TEM) images of Co‐CCN and Co‐ACN catalysts. The inset shows the corresponding SAED images, d, e) HAADF‐STEM image of Co‐CCN and Co‐ACN catalysts, f, g) EDS mapping images of the Co‐CCN and Co‐ACN catalysts.

Amorphous materials have attracted significant attention due to their highly unsaturated surface sites and uniform catalytic centers within chemical and structural environments,^[^ ^9^ ^]^ making them not only model catalysts but also ideal platforms for single‐atom loading.^[^ ^11^ ^]^ **Figure** **1**a illustrates the synthesis procedures for Co‐ACN and Co‐CCN. Through thermal treatment of CCN, we disrupted the intraplanar hydrogen bonding and weak interlayer van der Waals interactions, thereby obtaining uniformly amorphized ACN.^[^ ^10^ ^]^ Atomically dispersed Co atom catalysts anchored on ACN and CCN were fabricated by a top‐down strategy. Amorphization treatment barely alters the original microscopic morphology and elemental compositions (Figures S1, S2, Supporting Information). Similar Fourier‐transform infrared (FT‐IR) spectra and X‐ray photoemission spectroscopy (XPS) indicate the retention of the core structure of the heptane ring(Figures S3–S5, Supporting Information). The absence of carbon‐related D and G bands^[^ ^14^ ^]^ in the Raman spectra indicates that neither ACN nor Co‐ACN contains carbon‐like materials(Figure S6, Supporting Information). Meanwhile, both Co‐CCN and Co‐ACN exhibited ultrathin structure as well, without visible Co agglomerate (Figure 1b,c). Selective‐area electron diffraction (SAED) patterns exhibit a characteristic diffuse halo for Co‐ACN, in contrast to the sharp (002) diffraction rings of Co‐CCN (Figure 1b), indicating that the sample is amorphous, which is consistent with the X‐ray diffraction (XRD) results (Figure S7, Supporting Information).^[^ ^15^ ^]^ The structural distortion and atomic intercalation of Co atoms, along with the ultrasonic exfoliation, are likely to contribute to the enlargement of the surface area (Figure S8, Supporting Information), thereby providing additional reactive sites for PMS activation. High‐angle annular dark‐field scanning transmission electron microscopy (HAADF‐STEM) images of both Co‐CCN and Co‐ACN catalysts (Figure 1d,e) show isolated light spots anchored on the entire architecture without obvious aggregation of Co atoms. Energy‐dispersive X‐ray spectroscopy (EDS) mappings confirm the homogeneous distribution of carbon (C), nitrogen (N), and cobalt (Co) species on the carriers (Figure 1f,g). These results indicated that the atomically dispersed Co sites have been successfully anchored onto the ACN and CCN. The Co amounts in both Co‐ACN and Co‐CCN were detected by inductively coupled plasma‐atomic emission spectrometry (ICP‐AES), and the contents of Co were ≈3.2 wt% and 3.3 wt%, respectively.

**Figure 2:**
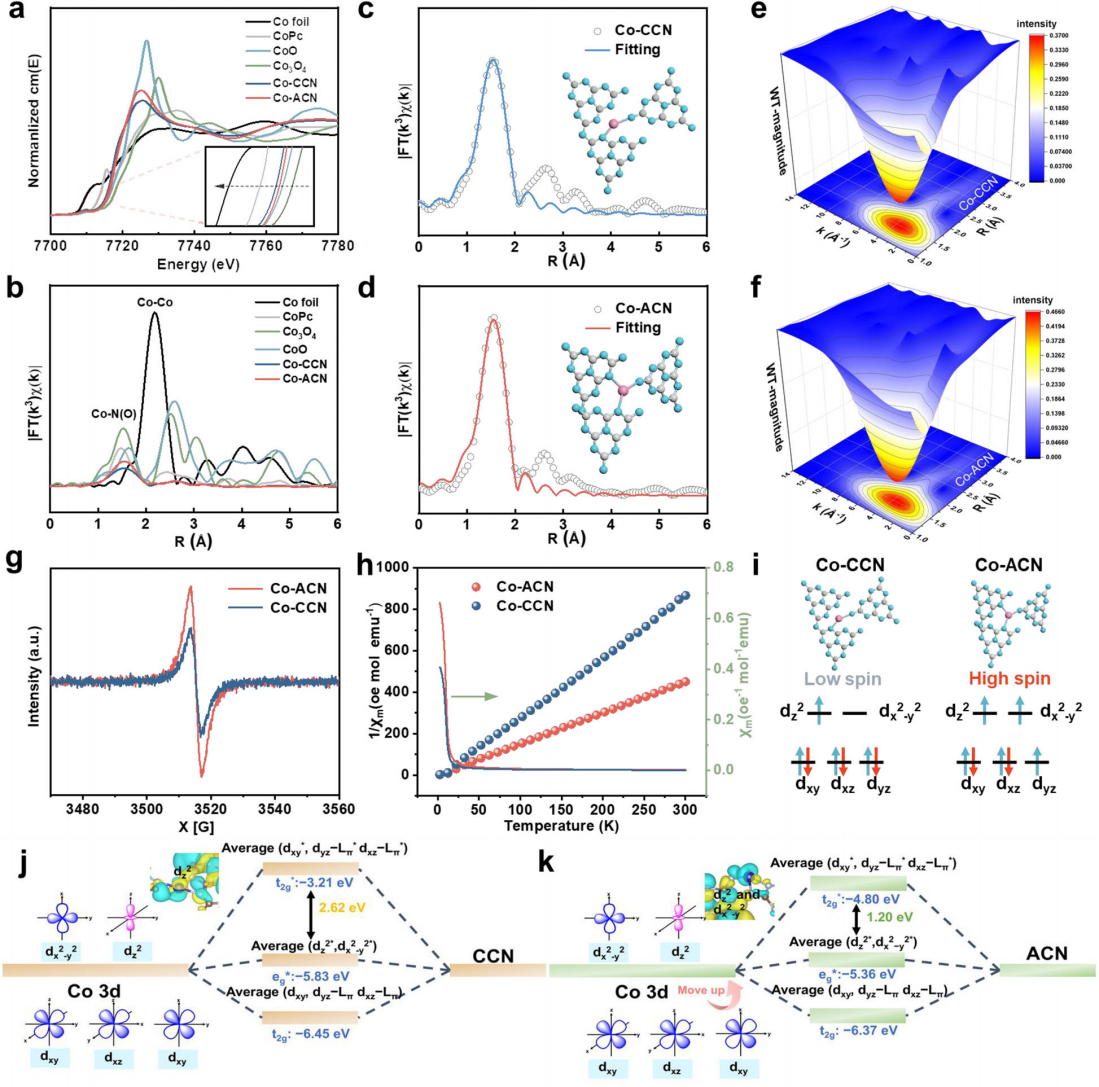
a) Normalized XANES survey spectra at the Co K‐edge of Co foil, CoO, Co_3_O_4_, CoPc, Co‐CCN and Co‐ACN, b) *k* ^3^‐weighted Fourier‐transform Co K‐edge EXAFS spectra of the samples, c, d) Co K‐edge EXAFS fitting curves of the Co‐CCN and Co‐ACN catalyst in the R space, respectively. Wavelet‐transformed *k* ^3^‐weighted EXAFS spectra of e) Co‐CCN and f) Co‐ACN, g) EPR spectra of Co‐CCN and Co‐ACN, h) χ_m_ and 1/χ_m_ plots of Co‐CCN and Co‐ACN, i) Schematic diagram of d‐orbital spin‐electron filling states. Orbital interaction analysis of j) Co‐CCN and k) Co‐ACN.

To determine the electronic structure and coordination environment of Co sites in Co‐CCN and Co‐ACN, X‐ray absorption near‐edge structure (XANES) and extended X‐ray absorption fine structure (EXAFS) spectroscopy at the Co K‐edge were performed. As shown in **Figure** **2**a, the absorption edge position of Co‐CCN and Co‐ACN is close to that of CoO but slightly lower, indicating that the valence state of Co is ≈+2. Notably, the adsorption edge energy of Co‐ACN shifts slightly to higher energy compared with Co‐CCN, suggesting the higher Co valence in Co‐ACN, as confirmed by the Co 2p XPS analysis. The *k* ^3^‐weighted Co K‐edge Fourier‐transformed EXAFS (FT‐EXAFS) spectra of Co‐CCN and Co‐ACN exhibit a dominant peak at ≈1.53 Å, which is mainly attributed to the scattering of Co‐N coordination (Figure 2b).^[^ ^16^ ^]^ Additionally, the intensity of the Co‐N peak progressively decreases from Co‐ACN to Co‐CCN, implying a reduction in the number of surrounding N atoms in the central Co atom.^[^ ^17^ ^]^ Moreover, no obvious Co‐Co coordination peak (2.18 Å) is observed compared with Co foil, manifesting that Co nanoparticles or clusters were not formed, in line with the results of HAADF‐STEM (Figure 1d,e) and XRD pattern (Figure S7, Supporting Information). The quantitative EXAFS fitting analysis provided precise coordination details for the two Co‐SACs, revealing Co‐N coordination numbers of 2.3 for Co‐CCN and 3.3 for Co‐ACN (Figure 2c,d and Table S1, Supporting Information). The atomic configurations of Co‐CCN and Co‐ACN, as well as the standard samples, were further investigated using wavelet‐transformed (WT)‐EXAFS (Figure 2e,f), which provides high resolution in both k‐space and R‐space. For the Co−O path, maximum intensities were observed at 6.6 and 6.4 Å^−1^ in CoO and Co_3_O_4_, respectively (Figure S9, Supporting Information). The Co−N path has maximized intensity at 3.9 Å^−1^. Similarly, an intensity maximum at 3.6 Å^−1^, corresponding to Co‐N coordination in both Co‐ACN and Co‐CCN, was observed. In contrast, the Co‐Co scattering signal at 6.9 Å^−1^ in Co foil, which was absent in both samples, further confirming the atomic dispersion of Co species. The best‐fit EXAFS results for Co foil, Co‐CCN, and Co‐ACN (Figures S10–S12, Supporting Information) support that cobalt predominantly exists in an atomic Co‐N form, anchored within both crystalline and amorphous C_3_N_4_ substrates.

The XPS was carried out to explore more information about the elemental compositions and chemical states of the catalysts. The similar binding energies of C 1s and N 1s in both ACN and CCN demonstrate that the amorphization treatment did not disrupt the core heptazine ring system, thereby confirming the retention of the short‐range atomic ordered structure in ACN (Figure S13, Supporting Information). The C 1s spectra of Co‐ACN and Co‐CCN display two prominent peaks at 284.7 and 288.2 eV, corresponding to C─C/C═C bonding and C─N═C bonding, respectively (Figure S13a,b, Supporting Information). In addition, the peaks at 286.3 eV are assigned to the sp^3^ C─N bond.^[^ ^10^, ^18^ ^]^ In addition, the three peaks related to C═N─C bonding (sp^2^‐hybridized N in the aromatic ring, 398.5 eV), N─C_3_ bonding (N atoms bounded to the tertiary carbon, 399.9 eV), and C─N─H bonding (amino groups carrying a hydrogen atom, 401.1 eV) were recorded in the N 1s region. A broad peak at 404.6 eV resulted from π–π^*^ excitations between the stacking interlayers. Compared to pure ACN and CCN, new characteristic peaks of Co─N (399.0 eV) bonds appeared in both Co‐CCN and Co‐ACN.^[^ ^19^ ^]^ (Figure S13c,d, Supporting Information) In addition, the Co 2p peak (Figure S13e,f, Supporting Information) can be fitted to two main satellite peaks.^[^ ^20^ ^]^ In the Co‐ACN nanosheets, the Co 2p spectra reveal two distinctive shakeup peaks at 797.0 eV and 781.3 eV, attributed to the 2p_1/2_ and 2p_3/2_ states, respectively. The peak at 781.3 eV is specifically associated with Co‐N coordination.^[^ ^20^ ^a]^ Notably, the binding energy of Co 2p_3/2_ in Co‐ACN shifts to a slightly higher value (by 0.2 eV) compared to Co‐CCN, indicating a relatively higher oxidation state of cobalt in the amorphous samples, consistent with XANES analysis.

Electron paramagnetic resonance (EPR) analysis (Figure 2g) revealed that the enhanced peak intensity in Co‐ACN compared to Co‐CCN is attributed to an increase in electron density within partially occupied d orbitals, indicating a significant proliferation of unpaired electrons on the Co atom.^[^ ^21^ ^]^ The temperature‐dependent magnetic susceptibility (M–T) measurement was conducted to reveal the electron spin configuration of Co‐N moiety in Co‐ACN and Co‐CCN molecules. Noteworthy, as shown in Figure 2h, the significant increase in the paramagnetic state of Co‐ACN is accompanied by an increase in the number of free electrons, and the number of unpaired electrons is further shown to be 1 for Co‐ACN and 3 for Co‐CCN by equation^[^ ^22^ ^]^ respectively, corresponding to the low and high spin configurations of Co (Figure 2i). From the above analysis, it could be seen that amorphization modulates the 3d‐orbital electronic structure of Co, and the spin state of crystalline Co‐CCN is low spin (LS, t_2g_^6^e_g_^1^), while the spin state of amorphous Co‐ACN is altered to high spin (HS, t_2g_^5^e_g_^2^). According to ligand field theory, the coordination of a metal center with weak field ligands results in a high spin state,^[^ ^23^ ^]^ as shown in Figure 2j,k.^[^ ^24^ ^]^ In the coordination compound formed by the Co center atom and ACN ligands, the high‐energy e_g_^*^ orbital and the low‐energy t_2g_^*^ orbital facilitated electron transfer, making it easier for electrons to transfer from the ligands to the metal center, causing the Co center to adopt a high spin state. Conversely, when the Co center atom coordinated with CCN ligands to form a coordination compound, the low‐energy e_g_^*^ orbital and high‐energy t_2g_^*^ orbital inhibited electron transfer. This further confirmed that the spin state of Co‐ACN was high spin, while the spin state of Co‐CCN was low spin. Further observation revealed that the energy gap between e_g_^*^ and t_2g_^*^ in Co‐ACN (1.20 eV) was smaller than that in Co‐CCN (2.62 eV). This indicated that Co‐ACN had a stronger electron transfer capability, enhancing the catalyst's reactivity and stability, which was beneficial for the rapid and stable degradation of pollutants.

### 2.2 Catalytic Performance of Fenton‐Like Reaction

**Figure 3:**
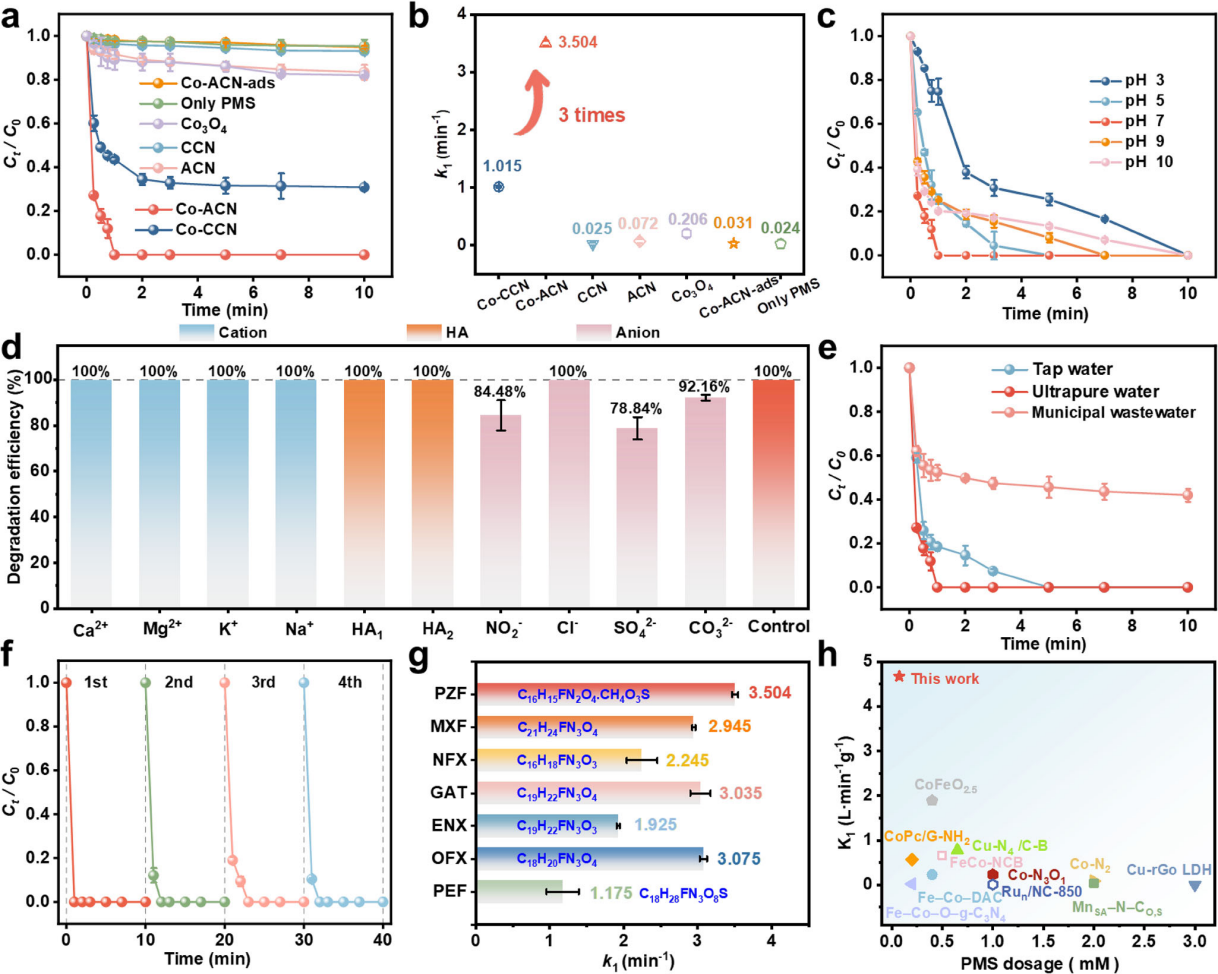
a) Degradation of PZF in different catalytic systems, b) The *k*_1_ values of PZF in different catalytic systems within 1 min, c) Effect of initial pH, d) Effects of adding anions, cations, or HA to Co‐ACN/PMS system on PZF degradation within 1 min, e) Degradation efficiency in different water qualities, f) The cyclic and regenerative properties of Co‐ACN/PMS system for PZF removal, g) The degradation *k*_1_ values of different pollutants in Co‐ACN/PMS system and structural formula of organics, h) Comparison of pollutant removal efficiencies of Co‐ACN with other recently reported catalysts vs PMS dosage. Reaction condition: [catalyst] = 0.1 g L^−1^, [PMS] = 75 µm, [Pollutants] = 10 µm, [anions and cation] = 2 mm, [HA_1_] = 0.22 µm, [HA_2_] = 0.44 µm, and initial pH = 7.0 ± 0.2.

The PZF was one of the common quinolones and frequently detected in pharmaceutical wastewater, which was considered a target contaminant for the degradation. **Figure** **3**a shows the removal rate of PZF for different systems. It showed the degradation of PZF by different catalysts under different conditions. When Co‐ACN alone was added to PZF, the concentration of PZF remained almost unchanged, indicating that Co‐ACN was not adsorptive. This suggested that Co‐ACN did not exhibit adsorption, and therefore, adsorption did not need to be considered in subsequent experiments. Within 10 min, less than 5.00% removal of PZF was achieved by PMS without catalysts. This result demonstrated that PMS alone could not degrade PZF. Figure 3b displayed the *k*_1_ corresponding to different systems, which were utilized to assess the catalytic efficiency of the catalysts. The degradation rates were 3.66% and 8.36% for CCN and ACN within 1 min, respectively, with corresponding *k*₁ values of 0.025 min^−1^ and 0.072 min^−1^. Upon incorporation of isolated Co atoms onto CCN to generate Co‐CCN, the degradation rate within 1 min reaches 56.38%, with a corresponding *k*₁ value of 1.105 min^−1^. Compared to other materials, the Co‐ACN/PMS system demonstrated a 100% degradation rate with the *k*_1_ value was 3.504 min^−1^ in 1 min, which was 3 times that of Co‐CCN. The results indicated that the amorphization led to a more disordered structure, increasing the specific surface area and providing more active sites for PZF degradation, thereby promoting electron transfer. In conclusion, the effect of Co‐ACN was better than other catalysts, which was chosen as the optimal material for subsequent PZF removal experiments. Finally, the PZF mineralization rate was also evaluated. Since the Co‐ACN/PMS system is primarily driven by the ^1^O_2_ mechanism, the total organic carbon mineralization is relatively low, ≈36.45% at 60 min (Figure S14, Supporting Information).

To investigate the universality of the Co‐ACN catalyst, the initial pH was varied. As shown in Figure 3c, when the pH of the solution was adjusted from 3 to 10, Co‐ACN exhibited a degradation rate of 100% within 10 min, demonstrating its capability to degrade PZF over a wide pH range. In the Co‐ACN/PMS system, the addition of anions/cations and humic acid (HA) (Figure 3d; Figure S15, Supporting Information) showed that different concentrations of HA and 2 mm cations had almost no effect on the removal efficiency of PZF. Only some anions preferentially reacted with ROS, resulting in a slight decrease in removal efficiency, but still able to degrade 100% of PZF within 10 min. Besides, different water quality conditions (Figure 3e) demonstrated that tap water did barely affect the degradation of PZF, but had a considerable impact on the degradation of pharmaceutical wastewater that did not contain PZF. This is possibly due to the catalyst preferentially degrading other active pharmaceuticals in the pharmaceutical wastewater. These results indicated that the Co‐ACN/PMS system possessed excellent anti‐interference capabilities under various water quality parameters. The cycling experiments demonstrated that after four cycles, the catalyst still achieved a 100% degradation efficiency within 3 min (Figure 3f), proving the stability of Co‐ACN. In addition, the XRD, XPS, and TEM analyses reveal that the structure and morphology of the Co‐ACN catalyst remain nearly unchanged before and after the catalytic reaction, thereby demonstrating its excellent stability (Figures S16–S18, Supporting Information). To evaluate the degradation effect of Co‐ACN on different quinolone antibiotics (Figure 3g; Figure S19, Table S2, Supporting Information), representative pharmaceutical such as pefloxacin (PEF), ofloxacin (OFX), enoxacin (ENX), gatifloxacin (GAT), norfloxacin (NFX), and moxifloxacin (MXF) were utilized. It was found that the above pollutants could be completely degraded rapidly, further demonstrating the versatility of the catalyst. Comparing the degradation ability of this system with the recently reported abilities of other systems (Figure 3h), it was found that its performance was much higher than that of other similar catalysts. This indicates that the catalyst is more stable and has excellent durability and versatility, possessing good prospects for practical application.

### 2.3 Mechanism Analysis

**Figure 4:**
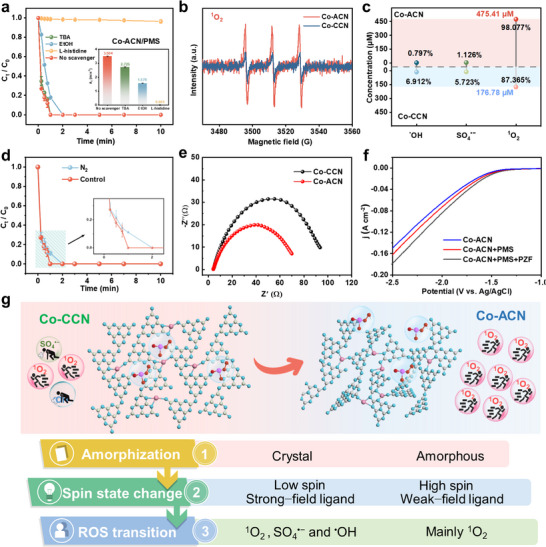
a) Influence of different quenchers on Co‐ACN/PMS system (Corresponding reaction rate constants *k_1_*), b) EPR spectra of TEMP‐^1^O_2_ (3 min), c) Different ROS yields in Co‐ACN/PMS and Co‐CCN/PMS systems, d) N_2_ was injected into the reaction system, e) Electrochemical impedance spectroscopy of Co‐ACN and Co‐CCN, f) Linear Sweep Voltammetry (LSV) analysis was performed on the Co‐ACN, g) Schematic diagram of the difference of ^1^O_2_ generated by different catalysts. Reaction condition: [catalyst] = 0.1 g L^−1^, [PMS] = 75 µm, [Pollutants] = 10 µm, [EtOH, TBA, and L‐histidine] = 50 mm, and initial pH = 7.0 ± 0.2.

To determine the reaction mechanism of the Co‐ACN/PMS system and Co‐CCN/PMS system, the ROS responsible for PZF degradation in different systems were explored, and various quenchers were introduced into the reaction system. Ethanol (EtOH) was the quencher of ^•^OH and SO_4_^•−^, and the tert‐butanol (TBA) was used as a radical quenching agent for ^•^OH.^[^ ^25^ ^]^ The addition of TBA and EtOH had little effect on the degradation performance of the Co‐ACN/PMS system (**Figure** **4**a). However, EtOH had a significant impact on the Co‐CCN/PMS system (Figure S20, Supporting Information). These results indicated that, compared to the Co‐ACN/PMS system, the Co‐CCN/PMS system generated more SO_4_^•−^ and ^•^OH. L‐histidine was a specific probe and quencher for the detection of ^1^O_2_ and was often used for the characterization of ^1^O_2_.^[^ ^26^ ^]^ The addition of L‐histidine to the Co‐ACN/PMS and Co‐CCN/PMS system were found to cause almost negligible degradation of PZF, indicating that a large amount of ^1^O_2_ existed in the above system. Thus, the ROS in the Co‐ACN/PMS system was mainly ^1^O_2_, while for the Co‐CCN/PMS system was ^1^O_2_, SO_4_^•−^ and ^•^OH during the degradation of PZF. It is well known that Deuterium oxide (D_2_O) is commonly used to measure the role of ^1^O_2_ in degradation experiments, as the lifetime of ^1^O_2_ in D_2_O is 10 times higher than in H_2_O.^[^ ^27^ ^]^ Therefore, relevant experiments were conducted, and the results indicate that in D_2_O, the Co‐ACN/PMS system enhanced the degradation efficiency of PZF, with the corresponding *k*_1_ value of 4.464 min⁻¹ within 0.75 min (Figure S21, Supporting Information). Meanwhile, the effects of other nonradical pathways, including the electron transfer process (ETP)^[^ ^28^ ^]^ and high‐valent Co‐Oxo species,^[^ ^29^ ^]^ on PZF degradation were also evaluated, indicating that the contributions of ETP and high‐valent Co‐Oxo are negligible (Figures S22, S23, Supporting Information). In addition, the results obtained from the quenching experiments were further validated by EPR experiments. The 2,2,6,6‐tetramethylpiperidine (TEMP)‐^1^O_2_ assay showed no significant characteristic peaks for both catalyst systems without adding PMS. After adding PMS to the catalytic reaction for 1 min, three characteristic peaks with intensity ratios of 1:1:1 were observed, indicating the presence of active species ^1^O_2_ in both Co‐ACN/PMS and Co‐CCN/PMS system during the degradation process (Figure 4b; Figure S24, Supporting Information).^[^ ^30^ ^]^ Comparing the results at 3 min, it was found that the peak intensity in the Co‐ACN/PMS system was much higher than that in the Co‐CCN/PMS system. This indicates that ^1^O_2_ existed in both systems, but the concentration generated in the Co‐ACN/PMS system was significantly higher than that in the Co‐CCN/PMS system. The analysis of 5,5‐dimethyl‐2‐pyrrolidone‐N‐oxyl (DMPO)‐^•^OH and SO_4_^•−^ (Figure S25, Supporting Information) showed that the Co‐CCN/PMS system exhibited prominent characteristic peaks after 1 min of reaction, whereas the peaks in the Co‐ACN/PMS system were much weaker. Comparing the characteristic peaks at 3 min, it was evident that the peaks in the Co‐CCN/PMS system were more pronounced and higher, indicating that this system generated substantial amounts of ^•^OH and SO_4_^•−^ during the degradation process compared to the Co‐ACN/PMS system.^[^ ^31^ ^]^ Therefore, the EPR results confirmed that the ROS in the Co‐ACN/PMS system primarily consisted of ^1^O_2_, whereas the ROS in the Co‐CCN/PMS system was included which was consistent with the results from the quenching experiments.

To perform quantitative analysis experiments of free radicals, Terephthalic acid (TPA) was used as a detector of ^•^OH, and p‐hydroxybenzoic acid (HBA) was used to detect SO_4_^•−^. The SO_4_^•−^ was used to determine the peak area of the detector by high‐performance liquid chromatography (HPLC), and the corresponding concentration, as well as the concentration of the generated free radicals, was obtained after the establishment of a standard curve. With 9,10‐Anthracenediyl‐bis (methylene) dimalonic acid (ABDA) as ^1^O_2_ detector,^[^ ^32^ ^]^ it was analyzed by ultraviolet–visible spectroscopy (UV–Vis) spectrophotometer, and the corresponding scalar curve was established to obtain the concentration of ^1^O_2_ (Figures S26,S27, Supporting Information). Results in Figure 4c displayed that the Co‐CCN/PMS system produced 13.99 µm (6.912%) of ^•^OH, 11.58 µm (5.723%) of SO_4_^•−^, and 176.78 µm (87.365%) of ^1^O_2_. The Co‐ACN/PMS system generated only 3.87 µm (0.797%) of ^•^OH and 5.46 µm (1.126%) of SO_4_^•−^, but 475.41 µm (98.077%) of ^1^O_2_, indicating that ^1^O_2_ plays a major role in the degradation process. In contrast, the Co‐ACN/PMS system generated almost 100% ^1^O_2_, with its concentration being 2.7 times higher than that of the Co‐CCN/PMS system. Thus, the higher production of ROS and the almost 100% selectivity toward ^1^O_2_, along with the unique advantages of ^1^O_2_ (longer lifetime, wide pH tolerance, and higher electrophilicity toward electron‐rich compounds), are attributed to the enhanced degradation performance of Co‐ACN/PMS system. When N_2_ was injected into the system to eliminate the interference of O_2_ in the system, the degradation efficiency decreased slightly (Figure 4d), but the degradation was still completed within 10 min, further confirming that ^1^O_2_ was mainly derived from the decomposition of PMS. To explore the electron transfer characteristics of the catalyst and reaction system, electrochemical impedance spectroscopy (EIS) and linear sweep voltammetry (LSV) were performed.^[^ ^33^ ^]^ As illustrated in Figure 4e, Co‐ACN demonstrates a lower resistance compared to Co‐CCN, indicating its superior conductivity and enhanced electron transfer rate. Additionally, the current density in the LSV curves exhibited an increase upon the addition of PMS, followed by a further rise with the introduction of the pollutant PZF (Figure 4f; Figure S28, Supporting Information). The results demonstrated that a reactive complex was first formed between PMS and the catalyst, and the PZF molecules subsequently provided electrons, accelerating PMS activation and thereby degrading the pollutant. Based on the above results, compared with crystalline Co‐CCN, the Co‐ACN/PMS system produced almost 100% ^1^O_2_ due to the amorphous process. Therefore, Figure 4g illustrates the reasons for the difference in ^1^O_2_ generation caused by different catalysts. The amorphous process changed the strong and weak field ligands, resulting in the transformation of the central spin states of atomic Co. In this process, CCN acted as a strong field ligand, while ACN served as a weak field ligand. When combined with the atomic Co center, this caused the spin state of the Co center to shift from low spin in Co‐CCN to high spin in Co‐ACN, facilitating electron transfer during the reaction and accelerating the generation of a large amount of ^1^O_2_. Amorphous engineering eventually leads to ROS transformation in heterogeneous Fenton‐like reactions.

### 2.4 Theoretical Computations

**Figure 5:**
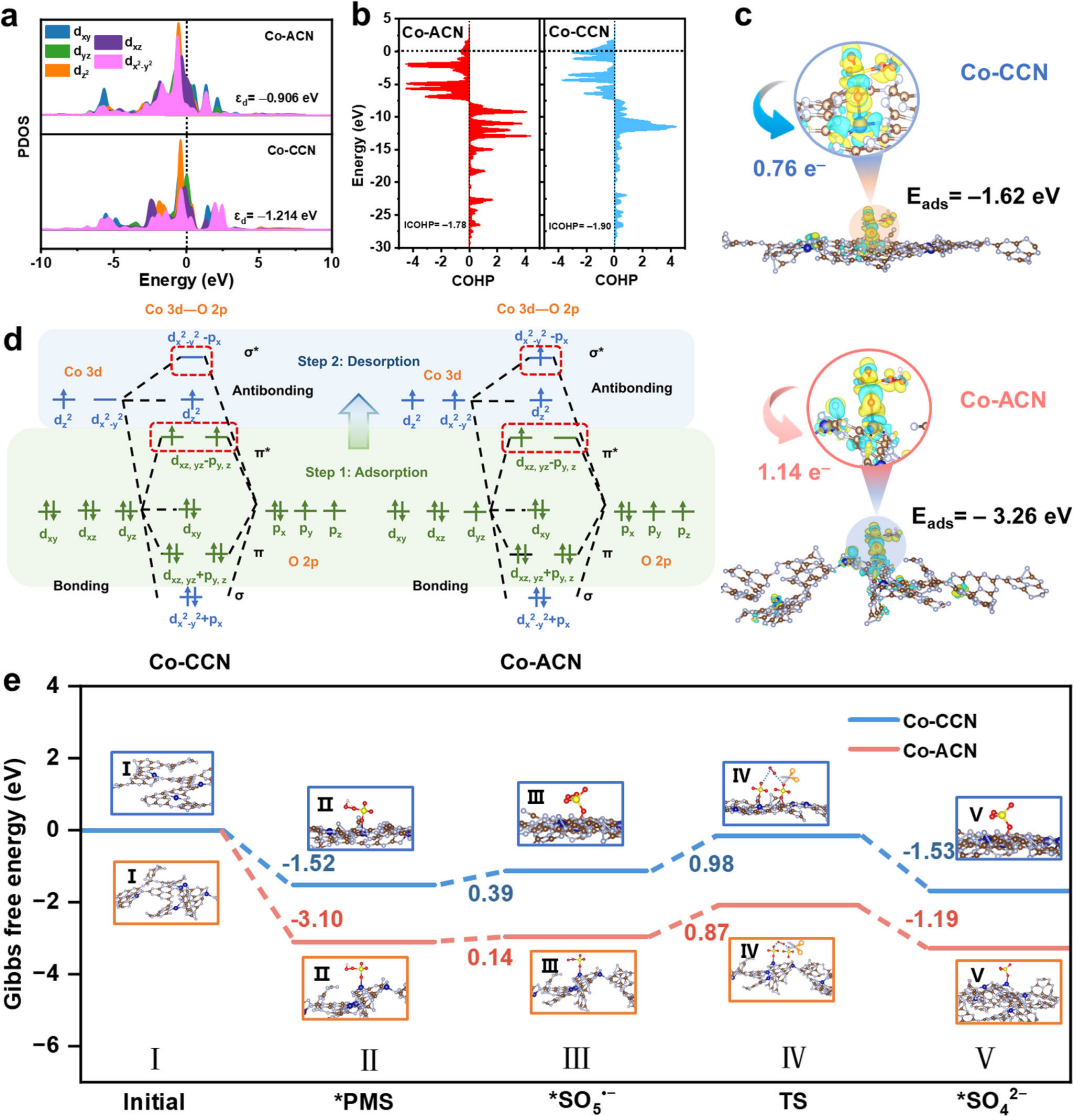
a)The. PDOS of Co‐ACN, Co‐CCN, b) Calculated COHP of Co‐ACN and Co‐CCN, c) The relevant adsorption energy and electrons transfer after PMS adsorption, d) The orbital interactions between HSO_5_^−^ and single‐atom Co catalysts, e) The Gibbs free energy of different coordination structures. (The white, red, yellow, blue, grey, and brown spheres represent the atoms of H, O, S, Co, N, and C, respectively).

The density functional theory (DFT) calculations were performed to deeply illustrate the improved PMS activation for Co‐ACN and Co‐CCN in **Figure** **5**. The projected density of states (PDOS) of Co 3d in Figure 5a showed that the d‐band center (Ɛ_d_) of Co‐ACN (Ɛ_d_ = −0.906 eV) was closer to the Fermi level than that of Co‐CCN (Ɛ_d_ = −1.214 eV), indicating higher electron activity and better PMS activation ability. Besides, it could be clearly observed in Figure 5a that the electron state of d_x_^2^_‐y_^2^ in Co‐ACN was more active than Co‐CCN, suggesting electrons were filled into d_x_^2^_‐y_^2^ and thus the spin state existed difference.^[^ ^34^ ^]^ The crystal orbital Hamilton population (COHP) results in Figure 5b revealed the strength of the bond for Co─N.^[^ ^35^ ^]^ The data of integrated COHP (ICOHP) for Co‐ACN (−1.78) was higher than Co‐CCN (−1.90), proving the enlarged antibonding filling and high spin state Co in Co‐ACN could be obtained. Thus, PDOS and ICOHP demonstrated the difference in the spin state between Co‐ACN and Co‐CCN, in which Co‐ACN belonged to a high spin and Co‐CCN was a low spin state. Because the d‐band center of Co 3d in the high spin state Co‐ACN was closer to the Fermi level than that in low spin state Co‐CCN, and the COHP value was lower than that of Co‐CCN, it declared that Co‐ACN and PMS had stronger chemisorption, interaction, and electron transfer capabilities. Subsequently, the energy of the adsorption between the PMS and the catalysts was discussed in Figure 5c.^[^ ^36^ ^]^ The Co‐ACN to HSO_5_^−^ possessed stronger adsorption energy (*E*_ads_ = −3.26 eV) than that of Co‐CCN (*E*_ads_ = −1.62 eV). The charge density difference (CDD) also clearly displayed 1.14 e^−^ transferred from Co‐ACN to HSO_5_^−^, more than that of Co‐CCN (0.76 e^−^ transferred to HSO_5_^−^). The XPS results of Co 2p indicate that the binding energies shift to lower values after the reaction, indicating that Co sites act as electron acceptors in the Co‐ACN/PMS systems, consistent with CDD trends (Figure S18, Supporting Information).^[^ ^37^ ^]^ Therefore, HSO_5_^−^ has a strong interaction and electron transfer with the high spin state Co‐ACN at first, further promoting PMS activation.

To profoundly elucidate the underlying mechanism of enhanced ^1^O_2_ generation in the Co‐ACN/PMS system, the orbital interaction for the process of adsorption and desorption between Co 3d of catalyst and O 2p of HSO_5_^−^ was presented in Figure 5d. It can be classified into two steps: i) The adsorption process: the Co 3d orbital was initially bonded to the O 2p of HSO_5_^−^ after the addition of PMS. Nevertheless, in contrast to the low spin state Co‐CCN, the π^*^ anti‐bonding orbital (d_xz‐yz_−p_y,z_) existed as an empty orbital in the high spin state Co‐ACN based on the bonding principle, further fewer electrons occupied in π^*^ anti‐bonding orbital. This indicated that when HSO_5_^−^ onto Co‐ACN, a stronger adsorption interaction in high spin state Co‐ACN can be acquired, which was consistent with adsorption energy and CDD results. ii) The desorption process: it could be observed that the high σ^*^ antibonding orbital (d_x_^2^_‐y_^2^ −p_x_) in high spin state Co‐ACN/HSO_5_^−^ was occupied after HSO_5_^−^ adsorption compared to the low spin Co‐CCN, indicating that, unlike the low spin Co‐CCN/HSO_5_^−^, a stronger repulsion interaction existed between Co 3d and O 2p. Therefore, after the adsorption of HSO_5_^−^, HSO_5_^−^ was more prone to desorption from the Co‐ACN. According to the above analysis, more SO_5_^•−^ can be generated due to the strong electron transfer and interaction when PMS is adsorbed on Co‐ACN. More importantly, the generated SO_5_^•−^ would more easily react to generate ^1^O_2_ due to decreased bonding between PMS and Co‐ACN based on the high spin state Coexistence of Co‐ACN. Finally, a possible pathway for the generation of ^1^O_2_ was proposed. Co‐ACN was inclined to adsorb PMS at the Co site, facilitating the oxidation of PMS to SO_5_^•−^ through the loss of an H atom. Due to the high reaction rate and low activation energy, SO_5_^•−^ can undergo rapid self‐reaction to generate ^1^O_2_.^[^ ^38^ ^]^ The free energy in Figure 5e further proved the process of PMS adsorption, SO_5_^•−^ and ^1^O_2_ generation in Co‐ACN was more likely to process compared to Co‐CCN, which was consistent with the above analysis.

## 3 Conclusion

In summary, thermal treatment was employed to disrupt the in‐plane hydrogen bonds and weak interlayer van der Waals forces in CCN, leading to uniform amorphization and thereby creating a distinctive anchoring platform for single‐atom cobalt. Based on a series of experimental investigations and DFT calculations, it was found that the electron rearrangement in the Co 3d orbitals shifted from the low spin state (LS, t_2g_^6^e_g_^1^, Co‐CCN) to the high spin state (HS, t_2g_^5^e_g_^2^, Co‐ACN), induced by substrate conformation change, is responsible for the greatly boosting ^1^O_2_ generation for Fenton‐like Reactions. The HS Co‐ACN optimized the d‐band center, boosted the electronic transfer, and weakened the interaction between Co 3d and O 2p orbitals of HSO_5_^−^, further promoting the generation of ^1^O_2_ with almost 100% selectivity, while Co‐CCN generated various ROS (^1^O_2_, ^•^OH and SO_4_^•−^). Under identical reaction conditions, the Co‐ACN/PMS system achieved complete degradation of PZF within 1 min, whereas the Co‐CCN system demonstrated a degradation efficiency of only 56.38%. The Co‐ACN/PMS system exhibited excellent resistance to interference and stability in the presence of common anions, cations, and organic substances in water, efficiently degrading pollutants under various water conditions and across a wide pH range. This study offers a novel approach to the atomic‐level design of catalysts, facilitating the selective generation of ^1^O_2_ and enabling the highly selective degradation of pollutants in wastewater.

## Conflict of Interest

The authors declare no conflict of interest.

## Supporting information



Supporting Information

## Data Availability

The data that support the findings of this study are available from the corresponding author upon reasonable request.
