# Supplementary material for: Amorphous Engineering Driving d‐Orbital High Spin Configuration for Almost 100% 1O2‐Mediated Fenton‐Like Reactions
Source: Adv Sci (Weinh). 2025 Apr 26;12(28):2503665. doi: 10.1002/advs.202503665 (PMC12302526; doi:10.1002/advs.202503665)
Supplement: Supplementary file 1 — Supporting Information [file ADVS-12-2503665-s001.docx]

**Supporting Information**

**Amorphous Engineering Driving D-Orbital High Spin Configuration for almost 100% ^1^O_2_-Mediated Fenton-like Reactions**

Juanjuan Qi^a,1^, Qian Bai^a,1^, Xiuhui Bai^b,^*, Hongfei Gu^b^, Siyue Lu^a^, Siyang Chen^a^, Qiangwei Li^a^, Xudong Yang^a^, Jianhui Wang^c^, Lidong Wang^a,^*

^a^ MOE Key Laboratory of Resources and Environmental Systems Optimization, College of Environmental Science and Engineering, North China Electric Power University, Beijing 102206, P. R. China.

^b^ School of Chemistry, Beijing Advanced Innovation Center for Biomedical Engineering, Key Laboratory of Bio-Inspired Smart Interfacial Science and Technology, Beihang University, Beijing, China.

^c^ Institute of Energy Resources, Hebei Academy of Sciences, Shijiazhuang 050081,China

*Corresponding Authors

E-mail address: xiuhuibai@buaa.edu.cn

wld@ncepu.edu.cn

^1^ Both authors (J. Qi, and Q. Bai) contributed equally to this work.

**Supporting Information** includes:

41 Pages

11 Texts

4 Tables

28 Figures

**Text S1.** **Chemicals and materials**

All chemicals and regents were analytical reagent (AR) or high-performance liquid chromatography (HPLC) grade. Urea (CH_4_N_2_O), Cobalt chloride hexahydrate (CoCl_2_·6H_2_O), Sodium chloride (NaCl), Magnesium chloride (MgCl_2_), Potassium chloride (KCl), Calcium chloride anhydrous (CaCl_2_), Ofloxacin (C_18_H_20_FN_3_O_4_), Potassium peroxymonosulfate (KHSO_5_), Sodium thiosulfate (Na_2_S_2_O_3_), L-Histidine (C_6_H_9_N_3_O_2_), Dimethyl sulfoxide (C_2_H_6_SO), Potassium carbonate (K_2_CO_3_), Pazufloxacin mesilate VETRANAL (C_16_H_15_FN_2_O_4_.CH_4_O_3_S) , Gatifloxacin (C_19_H_22_FN_3_O_4_) were obtained from Macklin Biochemical Technology Co., Ltd (China). Methanol (CH_3_OH), 5,5-Dimethyl-1-pyrroline N-oxide (C_6_H_11_NO), 2,2,6,6-tetramethylpiperidine (C_9_H_19_N), Pefloxacin Mesylate Dihydrate (C_17_H_20_FN_3_O_3_·CH_4_O_3_S·2H_2_O), Enoxacin (C_15_H_17_FN_4_O_3_), Norfloxacin (C_16_H_18_FN_3_O_3_), Moxifloxacin (C_21_H_24_FN_3_O_4_) were acquired from Shanghai Aladdin Biochemical Technology Co., Ltd (Shanghai, China). tert-Butanol (C_4_H_10_O) was obtained from Tianjin Damao Chemical Reagent Factory. Ethanol (C_2_H_6_O) was obtained from Tianjin Komeo Chemical Reagent Co., Ltd (China).Sulfuric acid (H_2_SO_4_), caustic soda (NaOH) were obtained from Tianjin Chemical Reagent Third Factory. Humic acid (HA) was obtained from Tianjin Beichen Fangzheng Reagent Factory.

**Text S2****.** **Characterization methods**

The surface morphology information of prepared catalysts was observed transmission electron microscopic (FEI Tecnai F20). A spherical aberration corrected high-angle annular dark-field scanning transmission electron microscope (HADDF-STEM) was applied to observe the Co single atom on the prepared catalyst. The X-ray diffraction (XRD, SHIMADZU XRD-6000) patterns was performed using an X-ray spectrometer equipped with a Cu-Ka radiation in the 2θ range of 10°-70° at a scan rate 5°/min. The structural information was obtained by Fourier transform spectrophotometer (FT-IR, TENSOR II, Bruker, Germany). N_2_ adsorption-desorption isotherms of the catalysts were measured using a Micromeritics ASAP-2460 analyzer. The specific surface area of the catalyst was estimated by Brunauer-Emmett-Teller (BET) method. Raman spectrum was acquired with a Jobin Yvon Raman spectrometer model HR800 with the excitation wavelength of 633 nm He-Ne laser line at room temperature. X-ray photon spectroscopy (XPS) measurements were taken in an ESCALab220i-XL (Thermo Scientific) using a monochromatic Al KR X-ray source. The X-ray absorption fine structure spectra data (Co K-edge) were conducted at BL11B station in Shanghai Synchrotron Radiation Facility (SSRF). The XAFS data were measured at room temperature in fluorescence excitation mode using a Lytle detector. The samples were prepared as plates of 10 mm diameter with 1mm thickness with the addition of PVDF powder as a binder. Total organic carbon (TOC) was determined with a TOC analyzer (Liqui TOCⅡ, Elementar Analysensysteme GmbH, Germany).

**Text S3. XAFS analysis**

The EXAFS data obtained were subjected to standard processing techniques using the ATHENA module within the IFEFFIT software suite. The specific steps of the process are as follows:

The EXAFS spectra that were collected were first processed by removing the post-edge background from the total absorption. This was followed by normalizing the data relative to the edge-jump. Next, the k^3^-weighted χ(k) data for the Co K-edge were transformed into real (R) space using a hanning window (d*k*=1.0 Å^-1^). This transformation was performed to distinguish the EXAFS signals from various coordination shells. To obtain the quantitative structural parameters around central atoms, least-squares curve parameter fitting was conducted by using the ARTEMIS module of the IFEFFIT software packages.

The following EXAFS equation was used:

In the equation, *S_0_^2^* represent the amplitude reduction factor*, F_j_(k)* is the effective curved-wave backscattering amplitude*, N_j_* is the number of neighbors in the *j^th^* atomic shell*, R_j_* represent the distance between the X-ray absorbing central atom and the atoms in the *j^th^* atomic shell (backscatterer)*, λ* represent the mean free path in Å*, σ_j_* is the Debye-Waller parameter of the *j^th^* atomic shell (variation of distances around the average *R_j_*) and *ϕ_j_(k)* is the phase shift (including the phase shift for each shell and the total central atom phase shift). The functions *F_j_(k)*, *λ* and *ϕ_j_(k)* were calculated with the ab initio code FEFF8.4.

**Text S4. Synthesis Methods of Catalysts**

*Synthesis of CCN and ACN.* Typically, 6.5 g of urea was subjected to heating at 550 ^o^C in a muffle furnace under a static air atmosphere for a duration of 4 hours, with a ramping rate of 8 ^o^C min^-1^. Subsequently, the sample was allowed to cool naturally to room temperature and then ground to yield crystalline graphitic carbon nitride (CCN). Amorphous carbon nitride (ACN) was obtained through the heat treatment of CCN at 620 ^o^C for 2 hours in an argon atmosphere, with a ramping rate of 5 ^o^C min^-1^.

*Synthesis of Co-CCN and Co-ACN.* 50 mg CCN or ACN was dispersed in 80 mL deionized water and stirred at room temperature for 2 hours. Then 1.5 mL CoCl_2_·6H_2_O aqueous solution (5 mg/mL) was added dropwise, followed by a continuous ultrasonic dispersion for 4h at room temperature. The resulting suspension was rapidly frozen in liquid nitrogen, and the products were collected by directly freeze-drying the suspension without further washing. The freeze-dried powder was uniformly placed in a quartz crucible and subjected to calcination in a 5% H₂/Ar atmosphere at 350 ^o^C for 2 hours. The obtained samples were thoroughly ground and then washed with 0.5 M H_2_SO_4_ to remove the residual Co nanoparticles, thereby yielding samples designated as Co-CCN and Co-ACN, respectively. To eliminate the interference of loading differences on performance and explore the intrinsic relationship between catalyst structure and activity, cobalt loading was strictly controlled within the threshold range for single-atom formation, ensuring the loadings of both catalysts are as similar as possible.

**Text S5. Catalytic Activity evaluation**

*Experimental methods for the degradation of PZF by the Co-ACN/Co-CCN*: Take 100 ml of ultrapure water in a 250 ml beaker, so there is no need for adsorption-resolution equilibrium, add 75 μL of PMS (100 mM) to the beaker, stir well, then use NaOH and H_2_SO_4_ to adjust the solution of the pH = 7, add 10 μL of PZF reserve solution (100 mM), and take several 1.5 mL liquid-phase vials to be used to take the samples. 100 μL of Na_2_S_2_O_3_ (100 mM) was added to the vials for depletion of PMS in the sample and left to mix well, 1ml of sample was taken from the beaker and filtered using 0.22 μm filter membrane and put into the vials. Then, 10 mg catalyst was added to start the timing, and the sample was taken using the same method at a fixed time point (15 s, 30 s, 45 s,1 min, 2 min, 3 min, 5 min, 7 min,10 min). PZF in the filtrate was determined by high-performance liquid chromatography (HPLC) (1260 Infinity II, Agilent, USA).

*Experimental method for free radical quenching in Co-ACN /PMS system*: The quencher was added prior to the addition of PZF using the method described above, and the rest of the steps remained unchanged

*Co-ACN /PMS degradation of PZF with anionic and cationic interference assays:* Add anions and cations before adjusting the pH of the solution by the above method, the rest of the steps remain unchanged.

*Cyclic degradation experiment*: After one PZF degradation cycle, the catalyst was collected using a 0.22 μm PTFE membrane and dried at 60 ℃. And the obtained powder was reused for the next degradation experiment under the same experimental conditions.

**Text S6. Electrochemical measurement**

Electrochemical measurements are conducted in a standard three-electrode system (CHI 660D), where the working electrode is an activator-coated carbon paper, the counter electrode is a platinum sheet, and the reference electrode is an Ag/AgCl electrode. To prepare the ink, 5 mg of catalyst is dispersed in 990 µL of isopropanol, followed by the addition of 10 µL of 5 wt% Nafion solution. After 20 minutes of sonication, a homogeneous ink is obtained. Next, 200 µL of ink is pipetted onto the carbon paper (1 × 1 cm^2^) for even coating and dried naturally at room temperature. Linear sweep voltammetry (LSV) experiments are performed in a 0.5 M Na_2_SO_4_ solution between -1 and -2.5 V vs Ag/ AgCl at a scan rate of 5 mV s^-1^. Electrochemical impedance spectra (EIS) data are collected with frequencies ranging from 10^4^ to 10^-1^ Hz. All electrochemical tests are conducted at room temperature.

**Text S7. Methods of analyzing contaminants**

An HPLC system consisting of an Agilent Venusil MP C18 column (4.6 nm, 5 μm) and a Wan Instruments UV3100 detector was used for the determination of PZF concentration. The mobile phase consisted of methanol and 1% formic acid solution with the volume ratio of 35:65, the flow rate of 1 mL/min, the injection volume of 10 μL, the temperature of the column temperature box at 31.1 ^o^C, and the detection wavelength of PZF at 244 nm. A standard curve was fitted with the concentration as the horizontal coordinate, and the ratio of the peak area *C_t_* of each concentration to that of the maximum concentration, *C*_0_ as the vertical coordinate, and the correlation coefficient was greater than 0.999.

**Text S8. The calculation of removal efficiency and reaction rate constant**

The degradation efficiency of PZF was calculated by the equation:

$$\text{Removal efficiency }(\%)\text{ = }(\text{C}_{0} \text{- }C_{t})\text{ / }\text{C}_{0}\times\text{100 \%}$$

The pseudo-first-order kinetics model:

$$k_{1}=-\text{ln}(C_{t}\text{/}\text{C}_{0})$$

where *C_0_* and *C_t_* represent the initial concentration of the pollutant and the concentration at a given reaction time t, respectively. $k_{1}$ (min^-1^) is the reaction rate constant.

**Text S9. Premixing experimental method**

Prior to the degradation of the pollutant, PMS and the Co-ACN catalyst were premixed for specific durations (6 min, 12 min). Subsequently, the pollutant (PZF) was introduced, and the timing commenced simultaneously. The reaction conditions were consistent with those described in the degradation experiment above.

**Text S9. PMSO consumption and PMSO_2_ generation experiment**

The experimental procedure follows the same protocol as the degradation experiment, with PMSO substituted for PZF. The concentrations of PMSO and PMSO_2_ are determined by high-performance liquid chromatography.

**Text S10. Computational details**

Ab initio calculations were performed with the periodic density functional theory (DFT) code Vienna ab initio simulation package (VASP).^[1]^ The exchange and correlation energy was calculated within the generalized gradient approximation (GGA) using the Perdew−Burke−Ernzerhof (PBE) functional.^[2]^ To include van der Waals forces, we added the D3 correction as implemented by Grimme et al.^[3]^ The electron-core interaction was described with the projector augmented wave (PAW) method.^[4]^ The electronic wave functions were expanded using a plane wave basis set with an energy cutoﬀ of 400 eV. All structures were relaxed until the residual forces on the free atoms were smaller than 0.02 eV/Å.

Meanwhile, the differential charge density of the two models were calculated based on the following equations:

$$\text{∆}\text{ρ}\text{(r) = }{\text{ρ}\text{(r)}}_{\text{Total}}\text{ - }\text{ρ}\text{(r)}_{C_{3}N_{4}}\text{ - }{\text{ρ}\text{(r)}}_{\text{Co}}$$

Where *ρ*(r)_Total_, $\text{ρ}\text{(r)}_{C_{3}N_{4}}$ and *ρ*(r)_Co_ represent the calculated charge density of total system, C_3_N_4_, and Co atom(s), that are under the same coordinates.

To evaluate the Co-ACN and Co-CCN adsorption ability for HSO_5_^–^, the adsorption energy was calculated whose expression was defined as Equation:

Eads = E (Co-CCN /Co-ACN-HSO_5_^–^)–E (HSO_5_^–^)–E (Co-CCN /Co-ACN)

The Gibbs free energies are defined as：

ΔG = E + E_ZPE_ – TS

where E, E_ZPE_, and TS refer to the total energy of the system, the zero-point energy, and the entropy.

Furthermore, Atomic Simulation Environment (ASE) package^[5]^ was employed to aid the model building. The partial charge densities were evaluated by the Bader charge analysis,^[6]^ the VASPKIT package^[7]^ and VESTA^[8]^ were employed to aid in the analysis of electronic structure.

**Text S11. Calculation of the number of unpaired electrons (n) and the effective magnetic moment (μ_eff_)**

The number of unpaired 3d electrons (n) of Co center is evaluated from the effective magnetic moment (*μ*_eff_) according to the following equation:

$$2.828\sqrt{\chi_{m}T}=\mu_{eff}=\sqrt{n(n+1)}$$

**Table S1.** Structural parameters extracted from the Co K-edge EXAFS fitting. (S_0_^2^=0.89)

| **sample** | **Scattering pair** | **CN** | **R(Å)** | **σ^2^(10^-3^Å^2^)** | **ΔE_0_(eV)** | **R factor** |
| --- | --- | --- | --- | --- | --- | --- |
| Co foil | Co-Co | 12 | 2.49 | 6.3 | 7.2 | 0.002 |
| Co-ACN | Co-N/C | 3.3 | 2.05 | 6.4 | 1.3 | 0.003 |
| Co-CCN | Co-N/C | 2.3 | 2.05 | 6.6 | 2.7 | 0.001 |

S_0_^2^ is the amplitude reduction factor; CN is the coordination number; R is interatomic distance (the bond length between central atoms and surrounding coordination atoms); σ^2^ is Debye-Waller factor (a measure of thermal and static disorder in absorber-scatterer distances); ΔE_0_ is edge-energy shift (the difference between the zero kinetic energy value of the sample and that of the theoretical model). R factor is used to value the goodness of the fitting.

**Table S2.** Test conditions for different pollutants.

| **Organic contaminants** | **Mobile phase** | | **Detection wavelength (nm)** | **Flow rate (mL×min^−1^)** |
| --- | --- | --- | --- | --- |
|  | **0.1% formic acid (%)** | **Acetonitrile**  **(%)** |  |  |
| **OFX** | 80 | 20 | 288 | 0.5 |
| **PEX** | 70 | 30 | 271 | 1.0 |
| **ENX** | 70 | 30 | 280 | 0.6 |
| **GAT** | 75 | 25 | 293 | 1.0 |
| **NFX** | 70 | 30 | 278 | 0.2 |
| **MXF** | 75 | 25 | 290 | 1.0 |

**Table S3.** Water quality indicators and testing methods for tap water.

| **Norm** | **Value** | **Detection method or instrument** |
| --- | --- | --- |
| pH | 7.53 | PHS-3C Electronic pH Meter |
| TOC (mg/L) | 5.35 | XZ-0135 Water Quality Analyzer |
| Conductivity (us/cm) | 498 | Conductivity meter |
| Turbidity (NTU) | 3.225 | Turbidimeter |
| DO (mg/L) | 8.42 | ZXRJY-I Dissolved Oxygen Meter |
| Total alkalinity (mmol/L) | 2.4 | Acid-base indicator titration |
| Hardness (mmol/L) as CaCO_3_ | 70.34 | EDTA titration (GB) |
| Cl^–^ | 64.18 | Chloride ion meter |
| Ammonia nitrogen(mg/L) | 0.39 | Sodium reagent photometry |

**Table S4.** Degradation efficiency of pollutants by different catalysts.

| Catalyst (g·L^-1^) | Pollutant (mM) | PMS (mM) | *k*_1_ (min^−1^) | K_1_ (L·min^−1^g^−1^) | References |
| --- | --- | --- | --- | --- | --- |
| **Co-ACN (0.1)** | **PZF (0.01)** | **0.075** | **3.504** | **4.672** | **This work** |
| CoPc/G-NH_2_ (0.02) | Phenol (0.01) | 0.2 | 0.2260 | 0.565 | ^[9]^ |
| Fe–Co–O–g-C_3_N_4_ (0.2) | SMX (0.04) | 0.2 | 0.085 | 0.02125 | ^[10]^ |
| CoFeO_2.5_ (0.2) | SMX (0.04) | 0.4 | 0.151 | 1.8875 | ^[11]^ |
| Co-N_2_ (0.2) | BPA (0.05) | 2 | 0.695 | 0.086875 | ^[12]^ |
| Cu-N4/C-B (0.1) | BPA (0.09) | 0.65 | 0.56 | 0.77538462 | ^[13]^ |
| Cu-rGo LDH (0.25) | BPA (0.09) | 3 | 0.046 | 0.00552 | ^[14]^ |
| Mn_SA_–N–C_O,S_ (0.2) | BPA (0.04) | 2 | 0.36 | 0.036 | ^[15]^ |
| Fe-Co-DAC (0.2) | BPA (0.08) | 0.4 | 0.23 | 0.23 | ^[16]^ |
| Co-N_3_O_1_ (0.1) | CIP (0.002) | 1 | 1.15 | 0.23 | ^[17]^ |
| FeCo-NCB (0.1) | BPA (0.05) | 0.5 | 0.659 | 0.659 | ^[18]^ |
| Ru_n_/NC-850 (0.02) | CIP (0.004) | 1 | 0.08 | 0.008 | ^[19]^ |


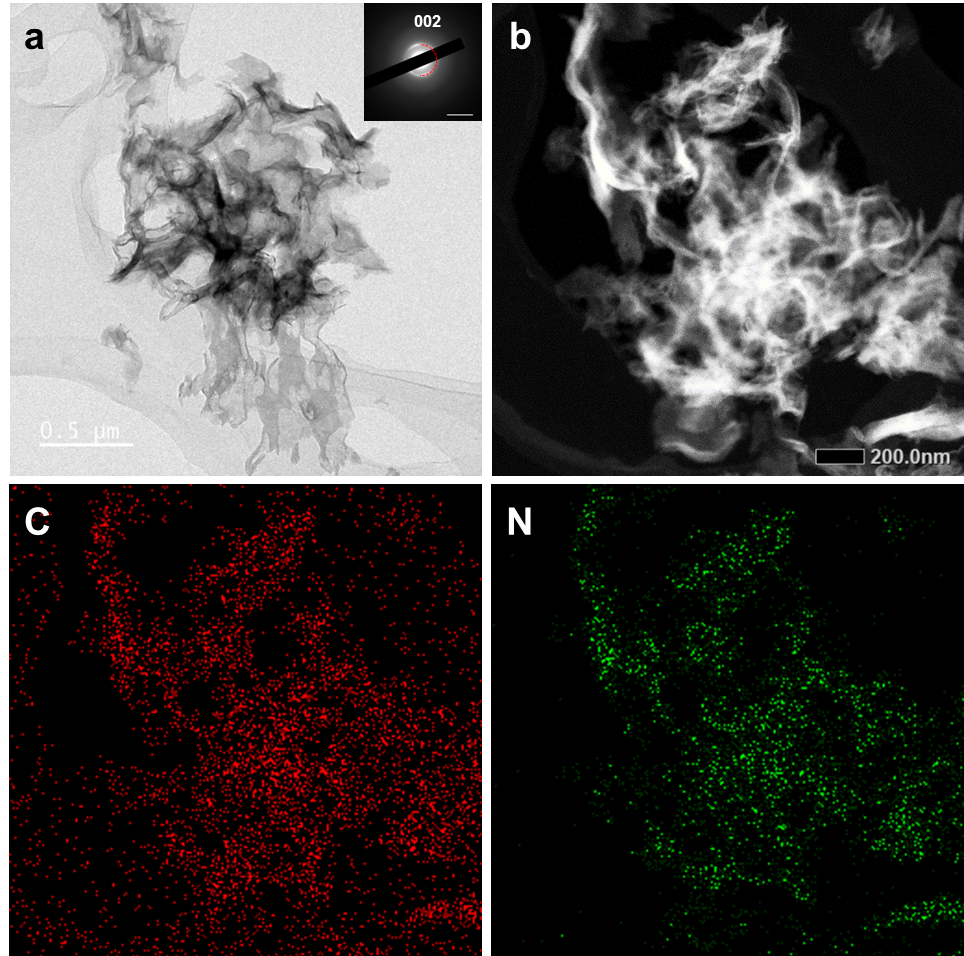


**Figure** **S1.** (a). TEM image of the CCN. The corresponding selected area electron diffraction (SAED) pattern also shows weak diffraction rings, indexed as the (002) peak of CCN. (b). STEM image of the as-prepared CCN and the corresponding EDS elemental mapping images.


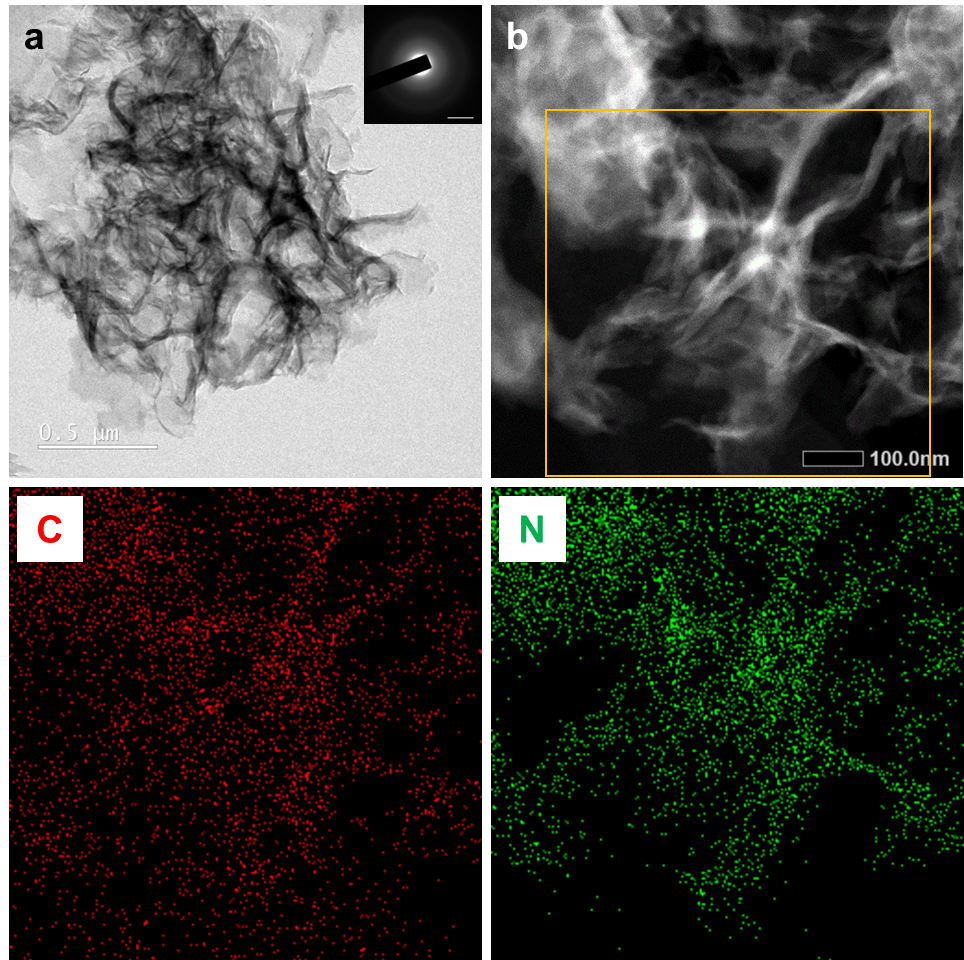


**Figure S2.** (a) TEM image of the ACN. The SAED pattern shows a diffuse halo, confirming the amorphous nature of the as-prepared ACN sample. (b) STEM image of the as-prepared ACN along with the corresponding EDS elemental mapping of the area highlighted in the yellow box.


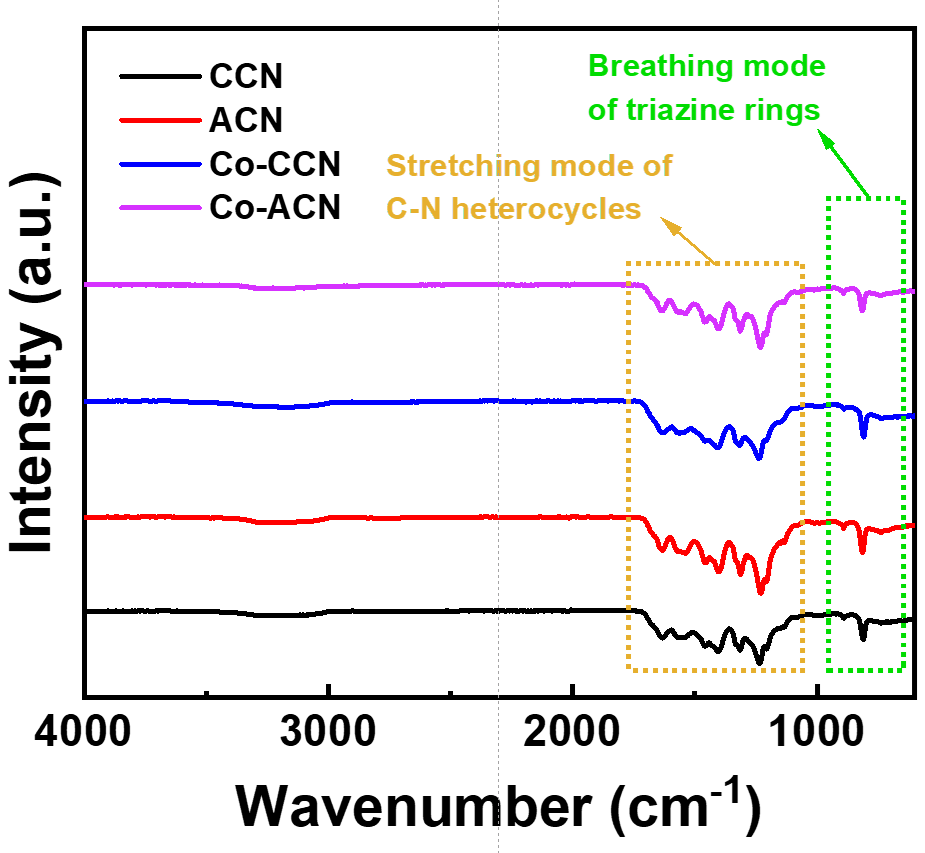


**Figure S3.** The FT-IR spectra of samples.

All catalysts display similar FTIR spectra, featuring peaks at 809 cm⁻¹ and within the 1200−1600 cm^-1^ range, which correspond to the breathing mode of triazine units and the stretching vibrations of C-N heterocycles in melon structures, respectively. These spectral characteristics suggest the retention of short-range atomic order in the amorphous materials.

**
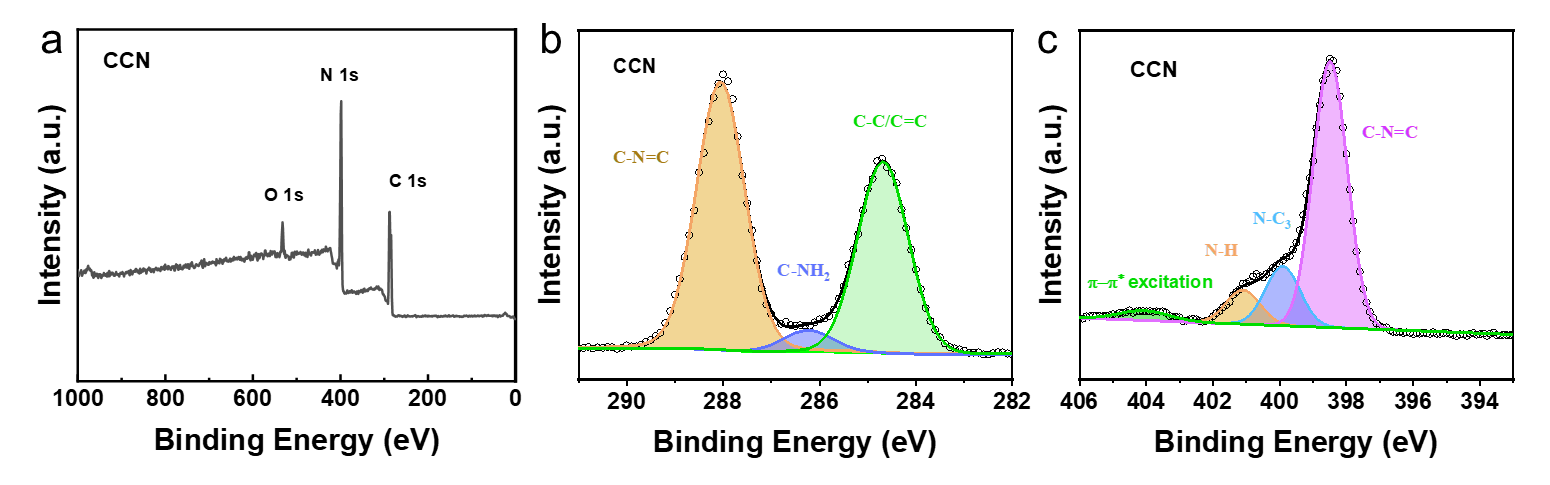
**

**Figure S4.** XPS spectra of CCN: (a) survey, (b) C 1s and (c) N 1s.


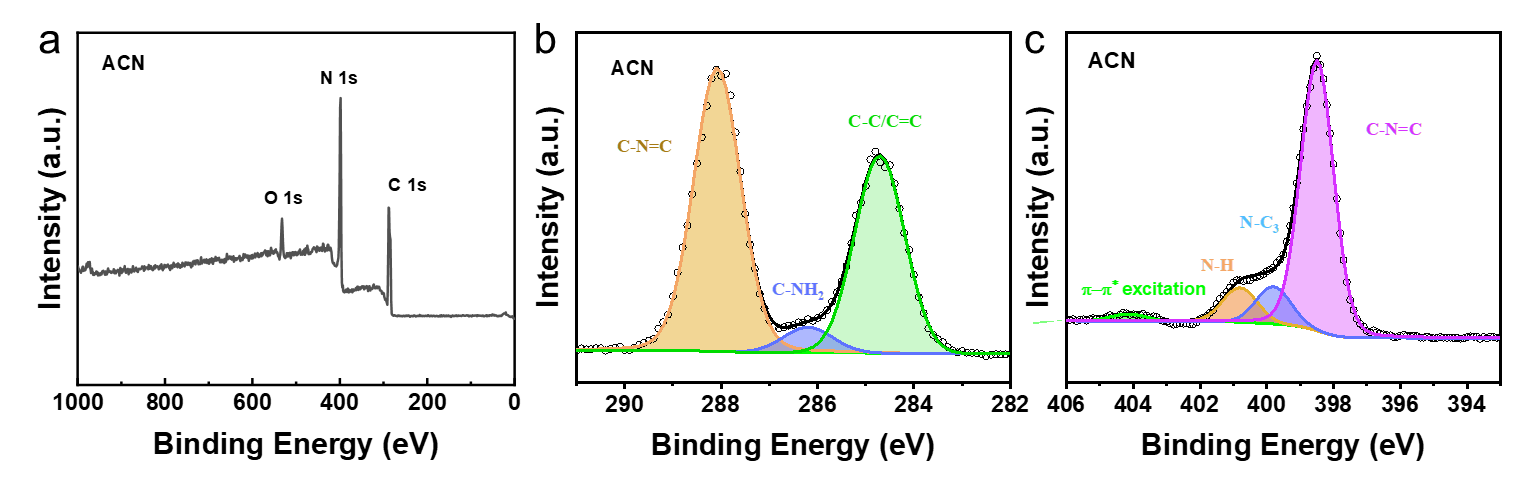


**Figure S5.** XPS spectra of ACN: (a) Survey, (b) C 1s and (c) N 1s.


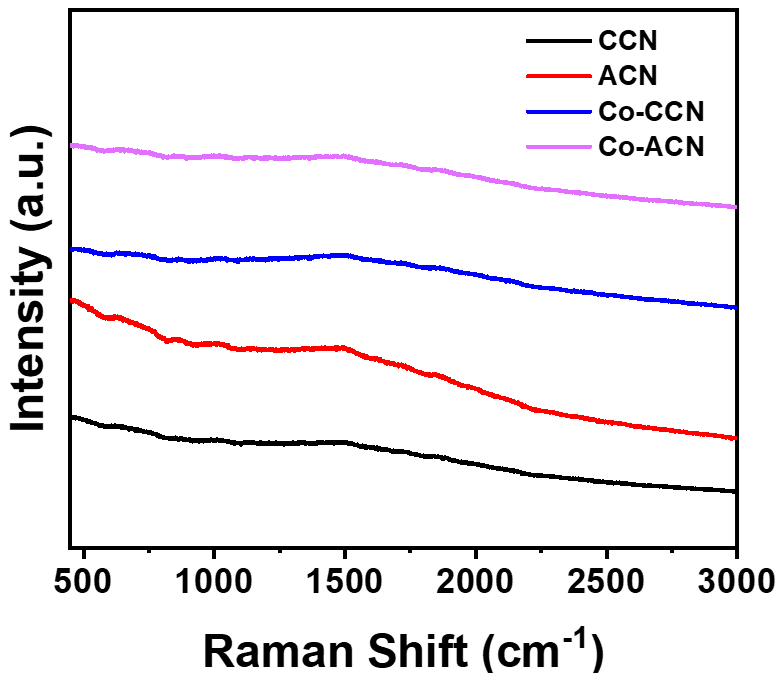


**Figure S6.** Raman spectra (633 nm) of CCN, Co-CCN, ACN and Co-ACN, respectively.


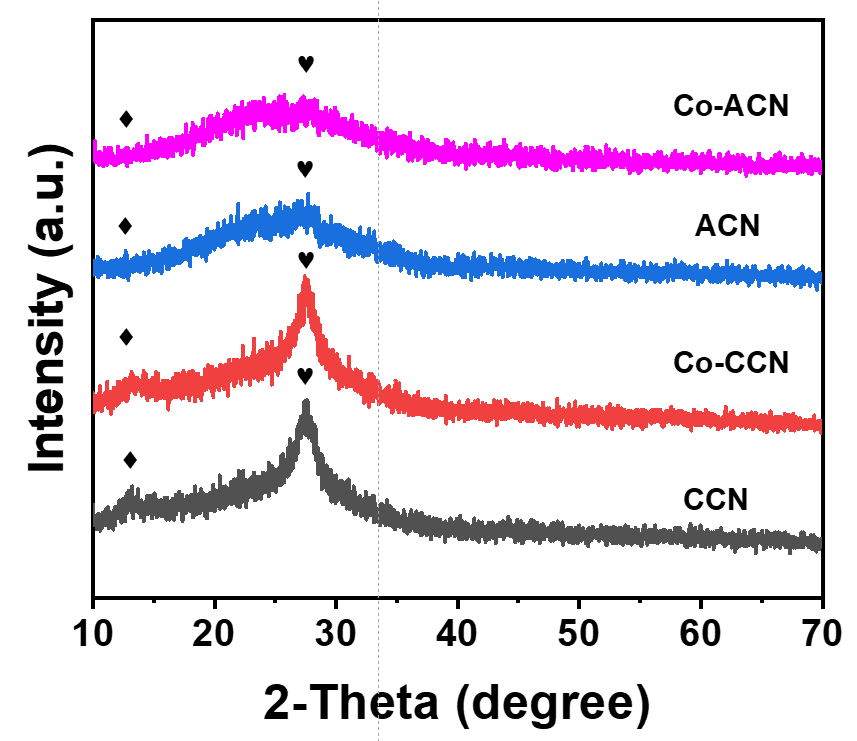


**Figure S7.** XRD patterns of CCN, Co-CCN, ACN and Co-ACN, respectively.


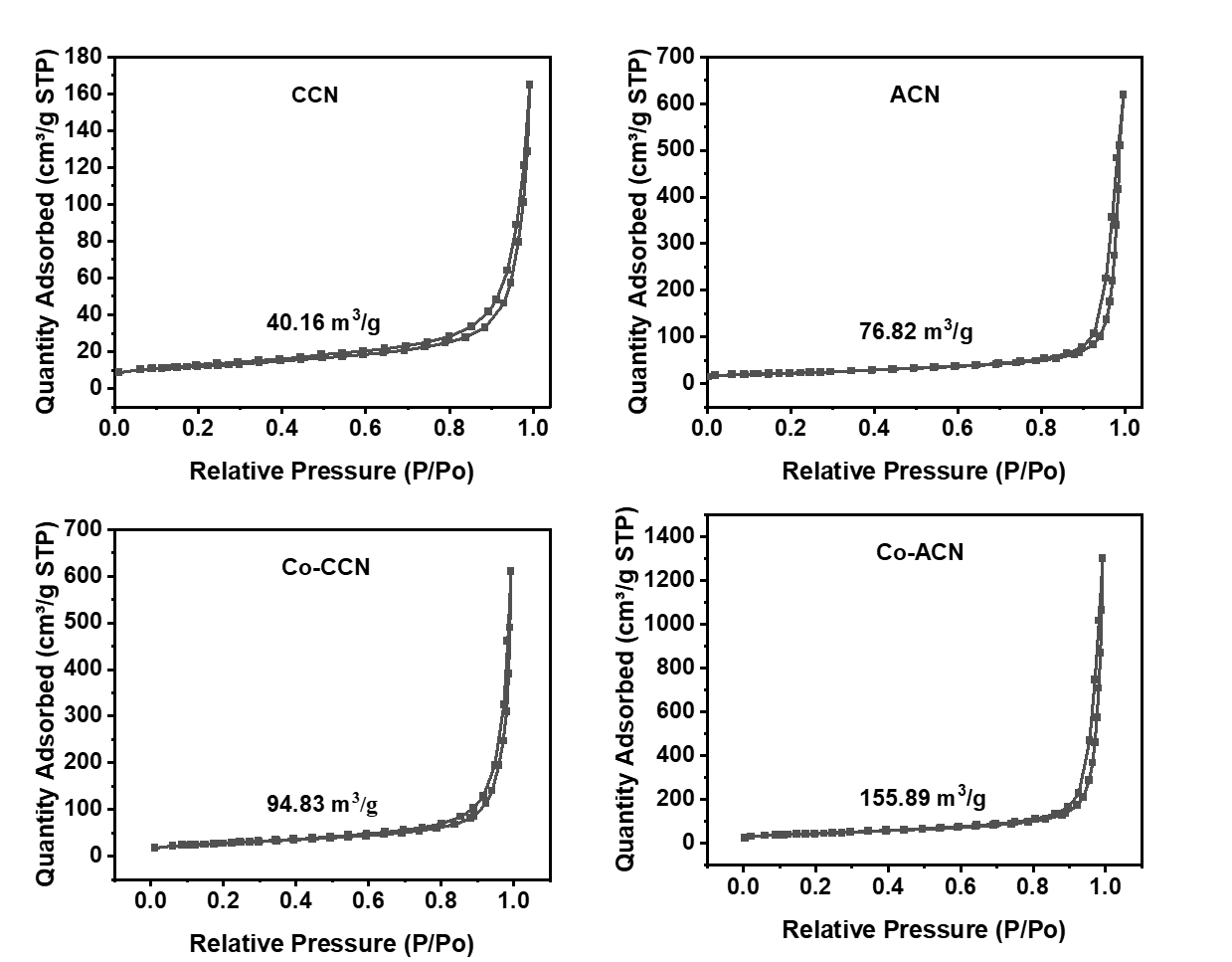


**Figure S8.** Isotherm nitrogen adsorption/desorption curves of CCN, ACN, Co-CCN and Co-ACN. The estimated specific surface areas ware 40.37 m^2^ g^-1^, 77.11 m^2^ g^-1^, 94.83 m^2^ g^-1^ and 155.89 m^2^ g^-1^, respectively.

The variation in the catalyst's specific surface area may be attributed to the following reasons:

**(ⅰ) Amorphization effects:** Heat treatment can significantly disrupt the long-range atomic ordering in both vertical and parallel directions to Crystal C_3_N_4_ (CCN) for uniform amorphization ^[20]^. During this process, the weak in-plane hydrogen bonds are broken, twisting the polymeric melon units due to the outward shift of NH_2_ groups. Consequently, the long-range atomic ordering in CCN becomes ruptured, whereas the strong covalent bonds in the melon units are preserved, resulting in amorphous C_3_N_4_ (ACN). Due to structural distortion in the ACN, its specific surface area increases by a factor of 1.3 compared to CCN^[21]^.

**(ⅱ) Atomic intercalation effects:** The Fu group^[22]^ found that even with a low Cu loading of 0.3 wt%, the specific surface area of Cu-TCN was significantly enhanced due to the atomic intercalation effect of Cu. Specifically, they observed a 1.6-fold increase in surface area for Cu-TCN compared to TCN without Cu loading. In our study, the loading of single-atom Co reached as high as 3.2 wt%, and the atomic intercalation effects is likely to exert a more significant influence on the specific surface area.

**(ⅲ) Effect of ultrasonic exfoliation:** During ultrasonic treatment, the intrinsic structural distortion of amorphous ACN, along with the intercalation of Co atoms, may facilitate substrate exfoliation, resulting in an increased surface area.


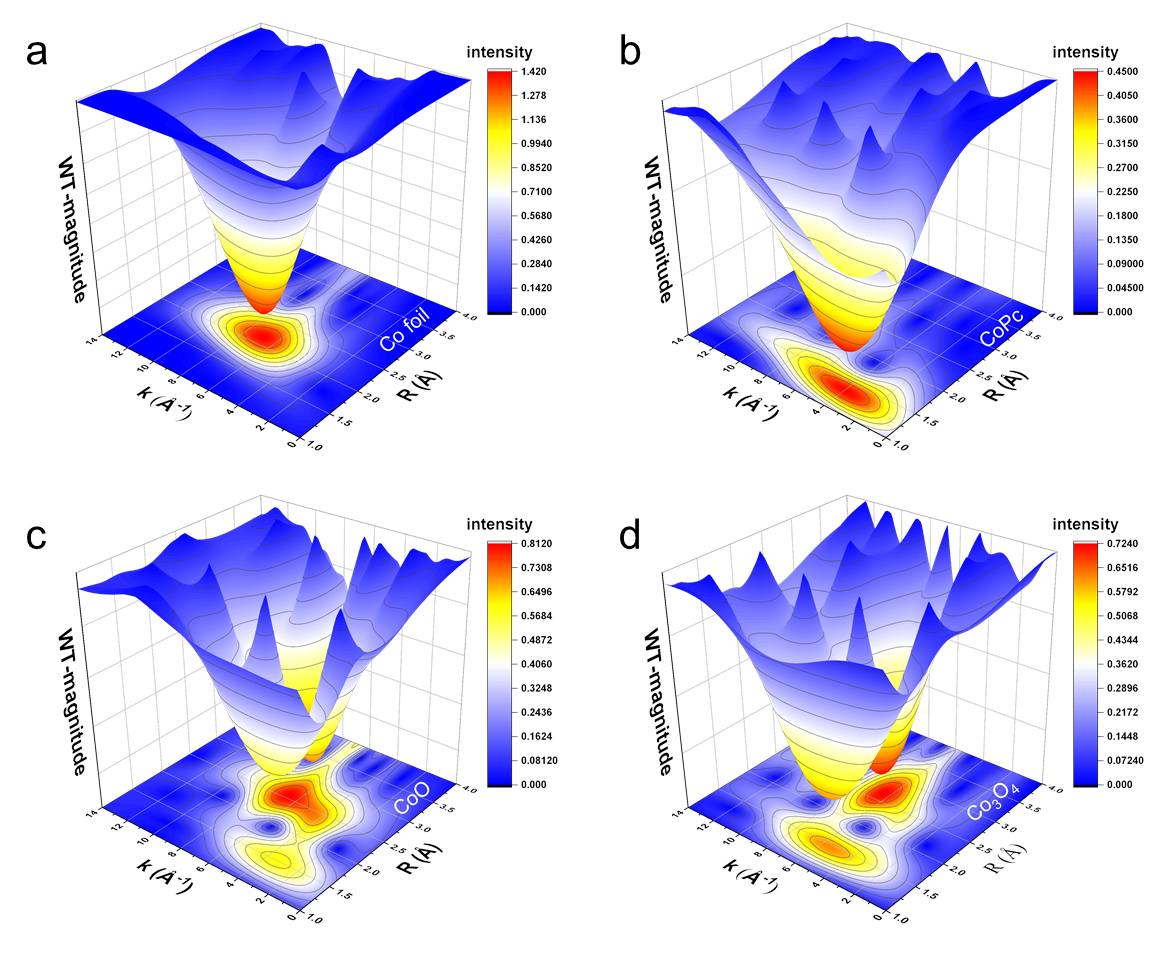


**Figure S9.** Wavelet-transformed *k*^3^-weighted EXAFS spectra of Co foil, CoPc, CoO and Co_3_O_4_.

**
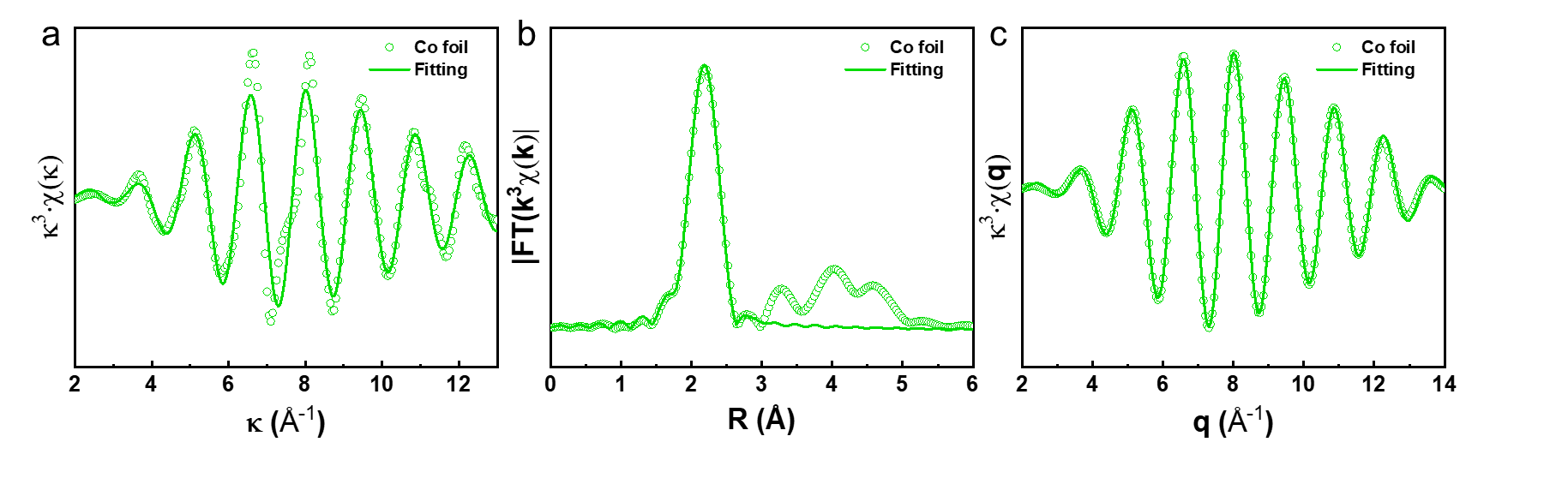
**

**Figure S10.** (a) k space, (b) R space and (c) q space EXAFS fitting curves of Co foil at Co K-edge.


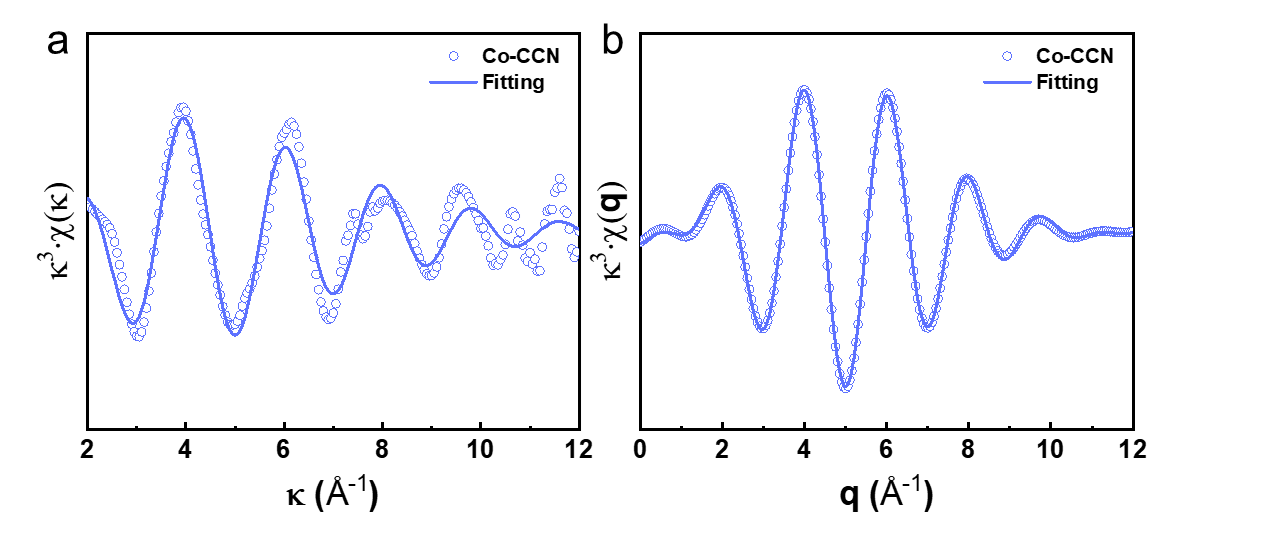


**Figure S11.** (a) k space, (b) q space EXAFS fitting curves of Co-CCN at Co K-edge.


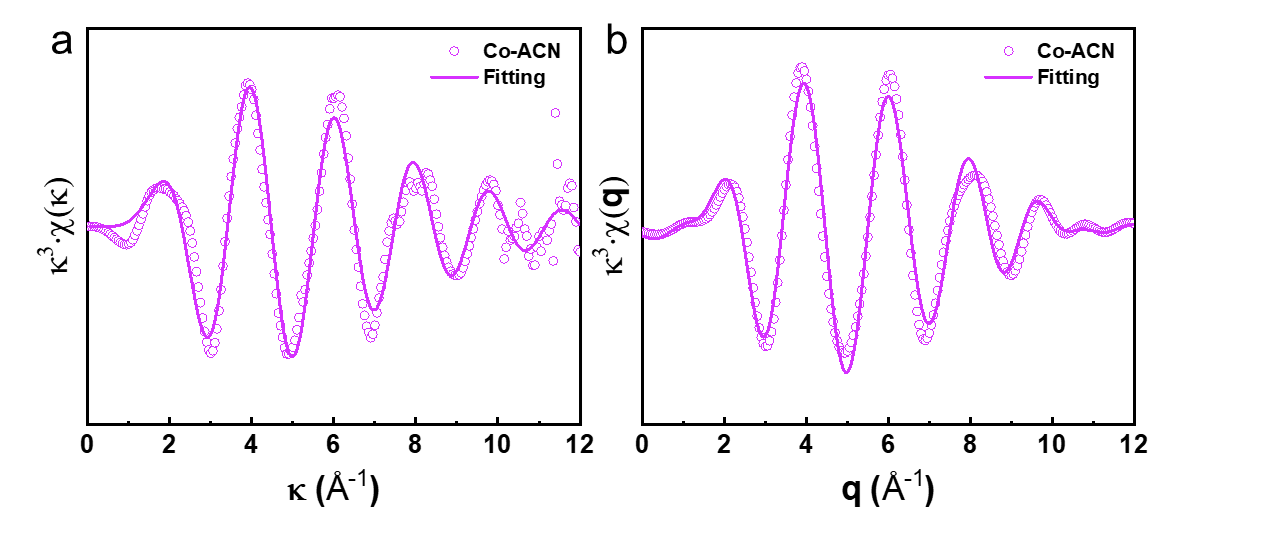


**Figure S12.** (a) k space, (b) q space EXAFS fitting curves of Co-ACN at Co K-edge.


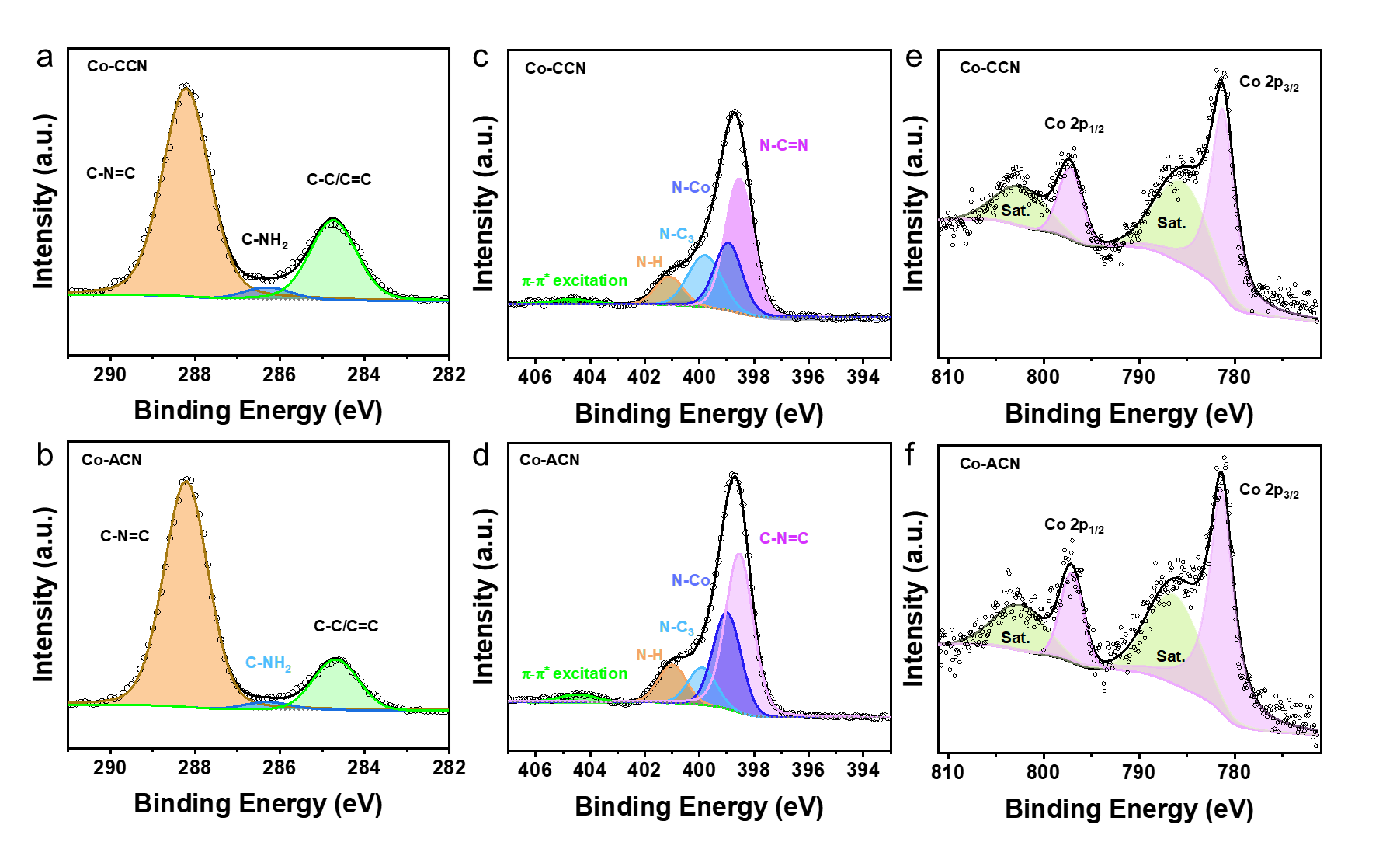


**Figure S13.** XPS spectra of Co-CCN and Co-ACN. The C 1s (a, b), N 1s (c, d) and Co 2p (e, f) region of Co-CCN and Co-ACN, respectively.

**Figure S14.** TOC removal rate during PZF degradation in Co-ACN/PMS system.


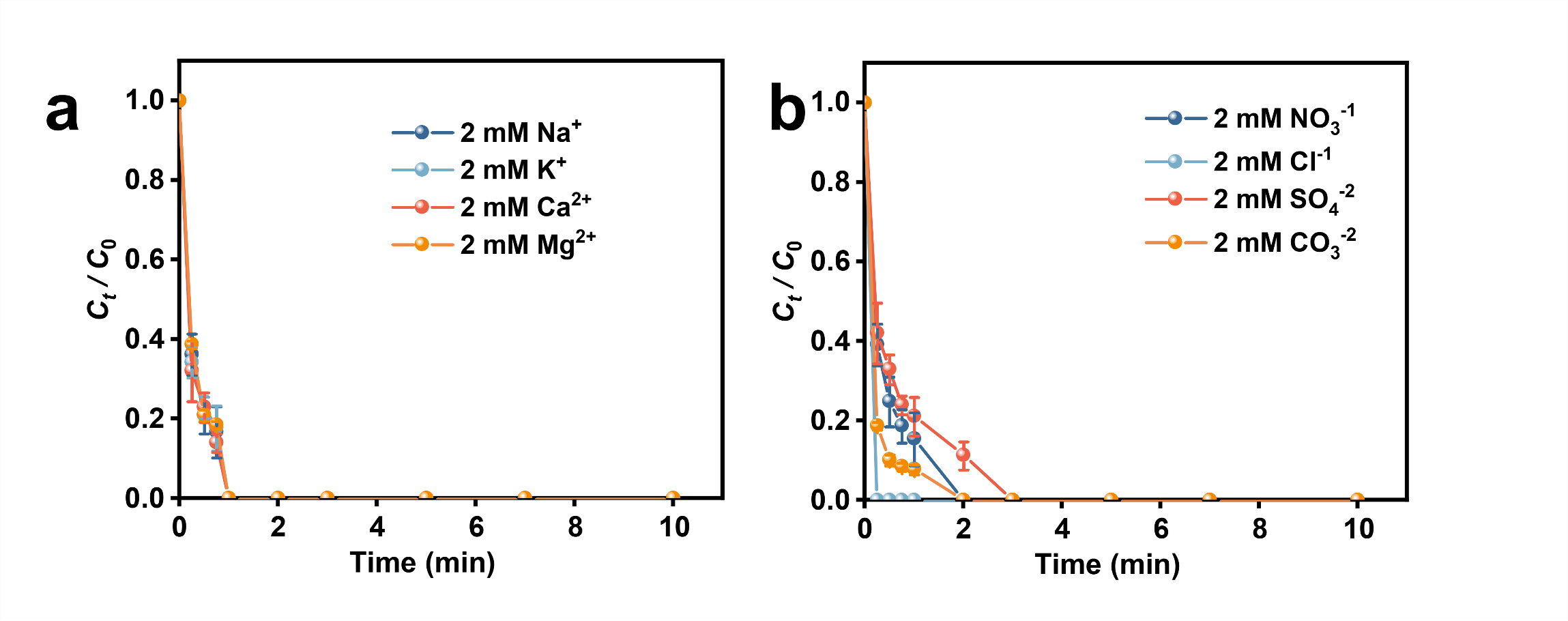


**Figure S15.** Degradation curves for various systems; influencing factors of: (a) anions, (b) cation. Reaction conditions: [catalyst] = 0.1 g L^-1^, [PMS] = 75 µM, [Pollutants] = 10 µM, [anions and cation] = 2 mM, and initial pH = 7.0 ± 0.2.


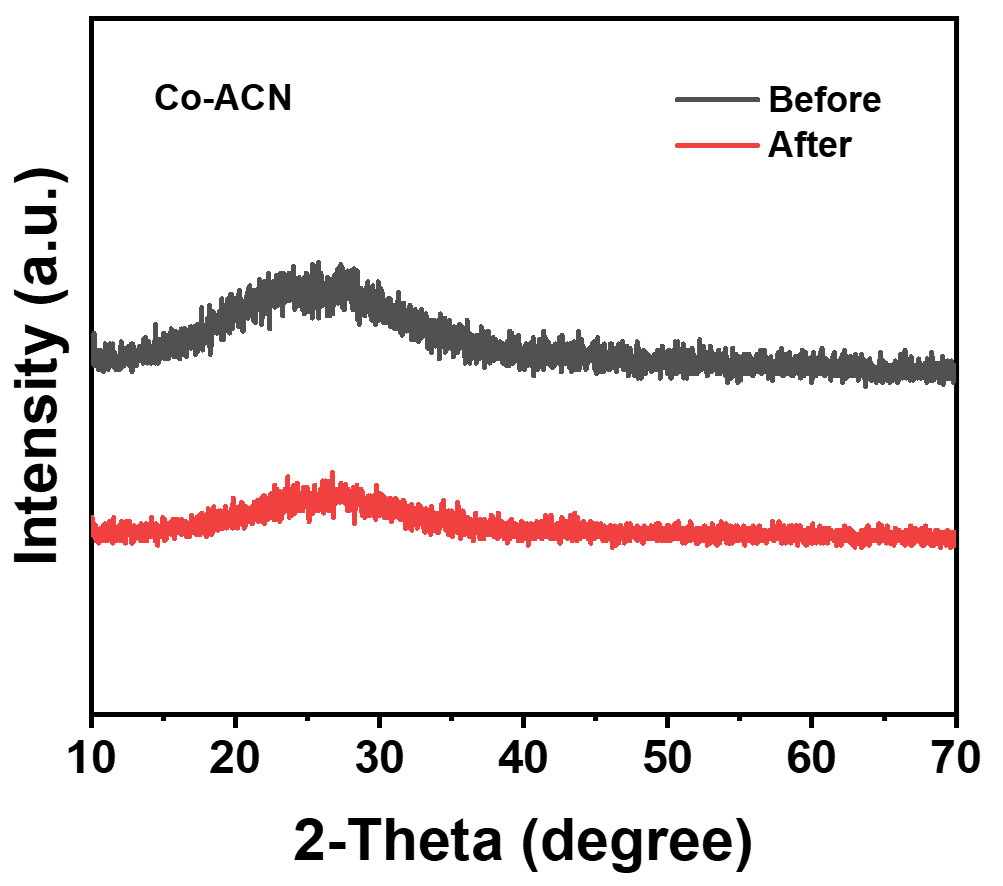


**Figure S16.** XRD patterns of Co-ACN before and after cyclic degradation reaction.


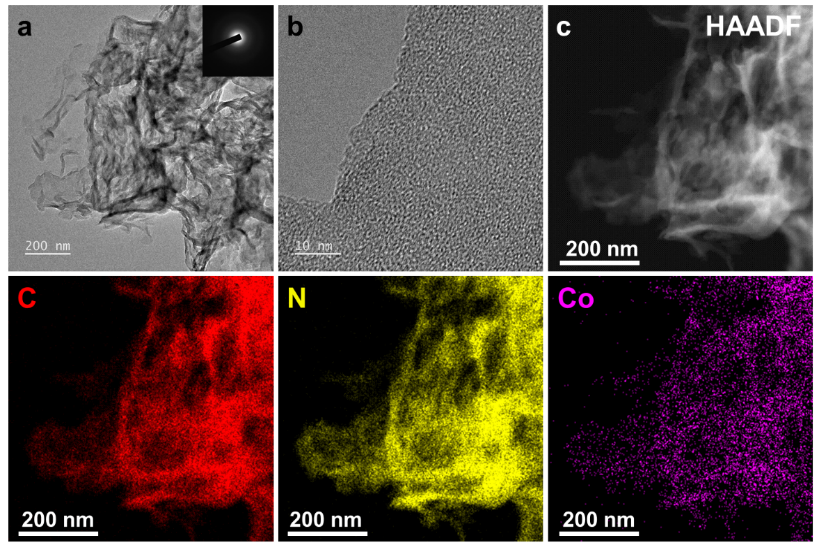


**Figure S17. Characterizations of Co-ACN catalysts after cyclic degradation reactions.** (a, b) TEM images of the Co-ACN catalyst, with the insets showing the corresponding SAED pattern. (c) HAADF image and the corresponding EDS mapping of the Co-ACN catalyst.


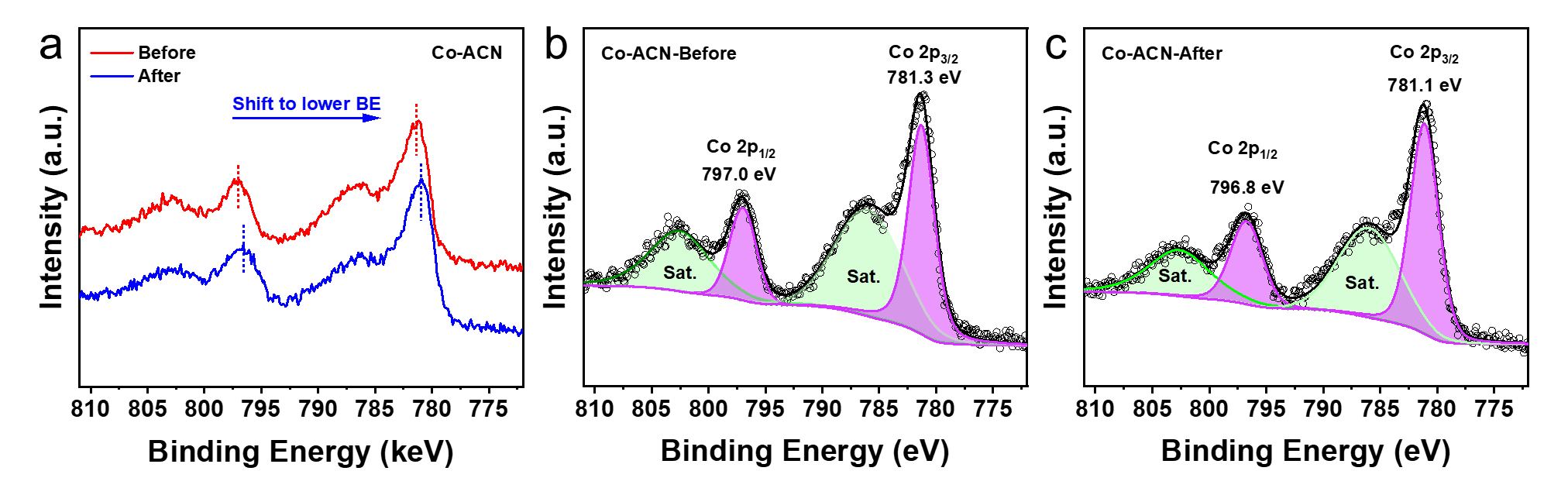


**Figure S18.** XPS analysis of the Co 2p fine spectra of Co-ACN before and after the reaction.


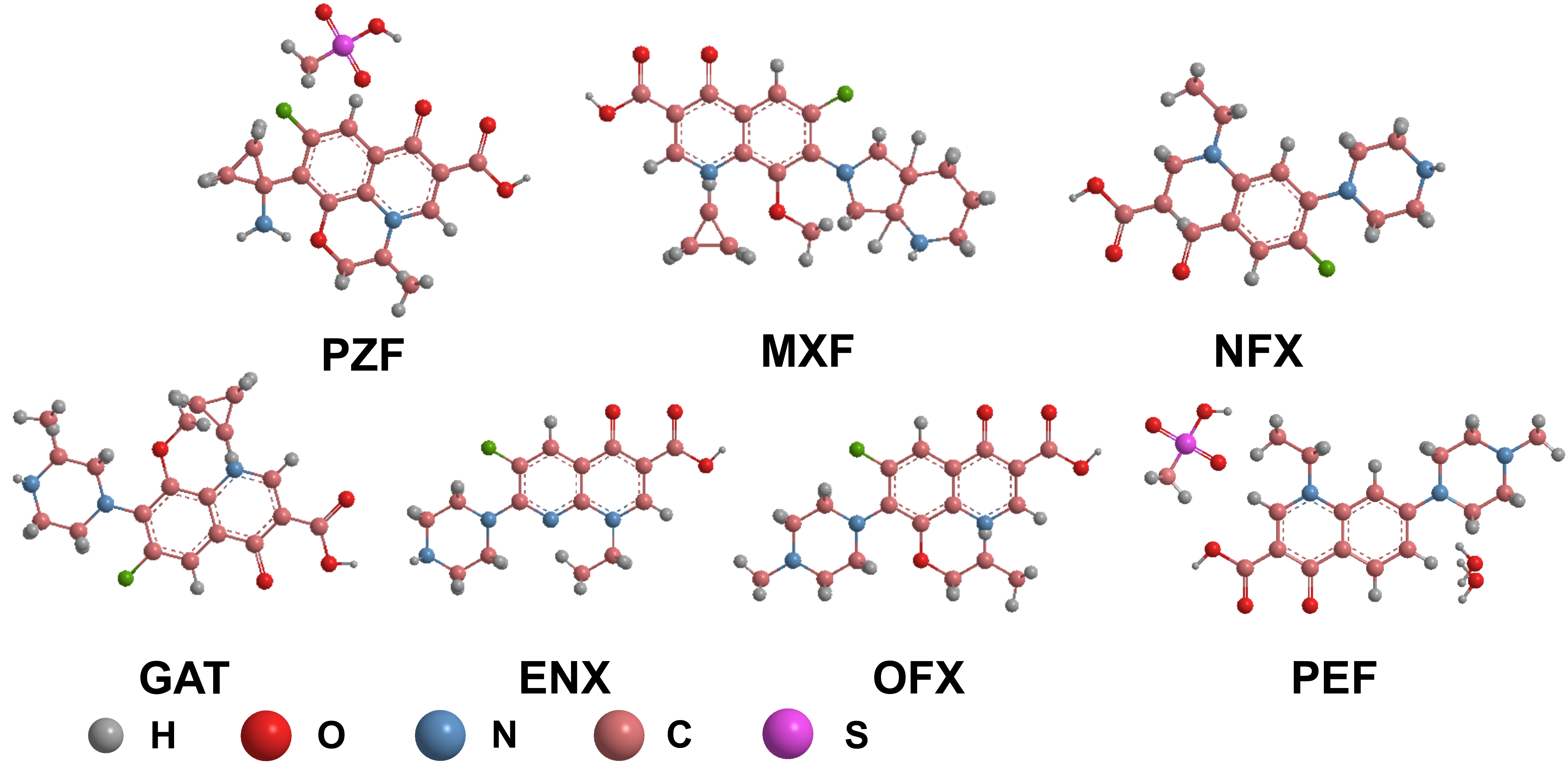


**Figure S19.** Structural formula of different organics.


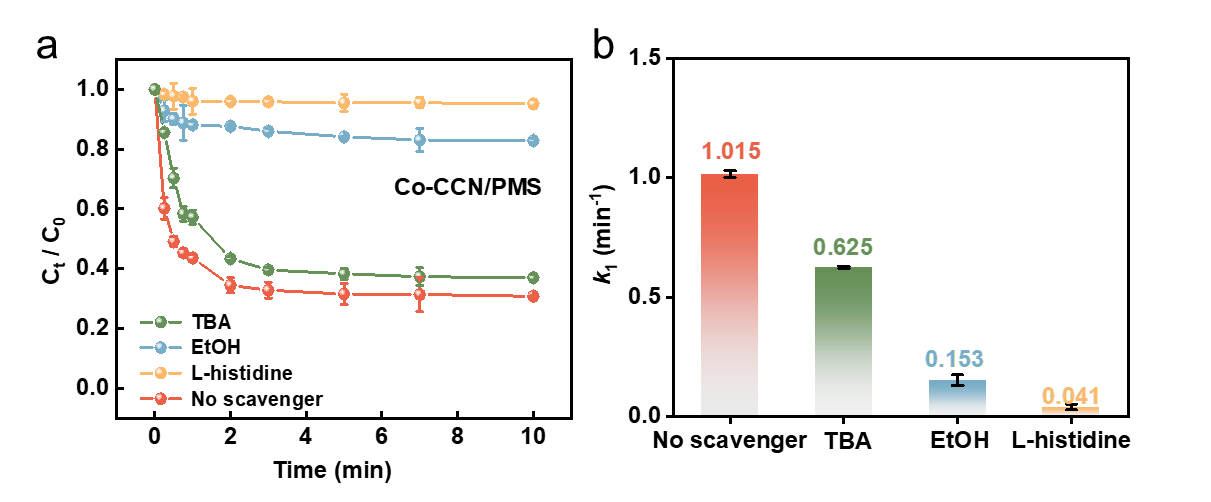


**Figure** **S20. (**a) Influence of different quenchers on Co-CCN/PMS system. (b) Corresponding reaction rate constants *k*_1_ within 1 min*.*


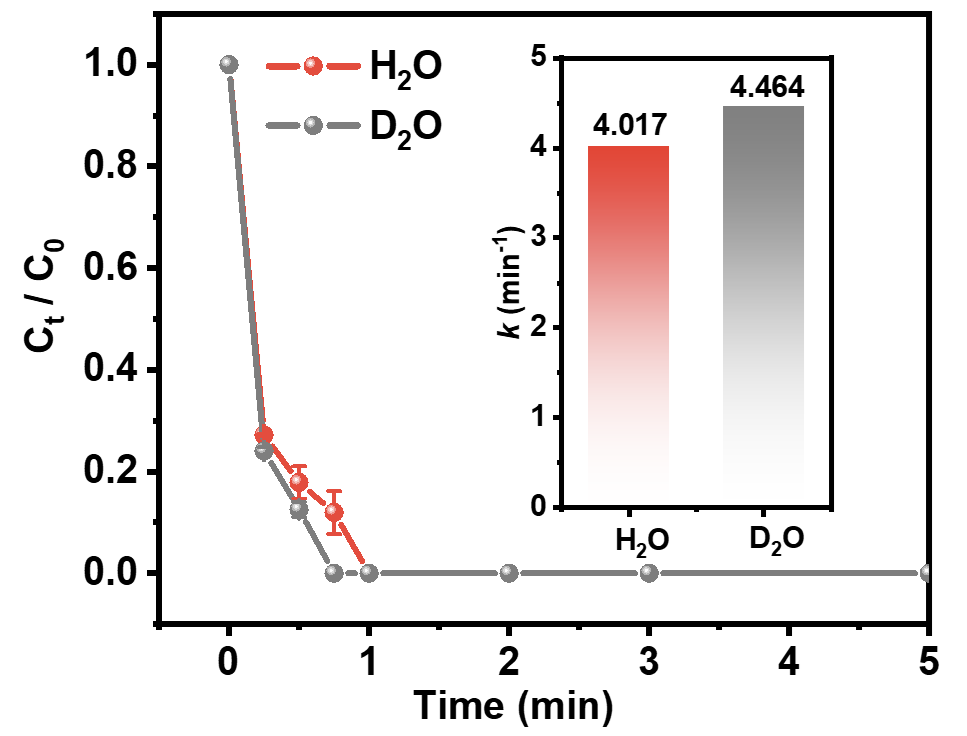


**Figure S21.** Degradation of PZF by Co-ACN/PMS system in D_2_O. The inset presents the *k*_1_ values for PZF degradation within 0.75 min. [Co-ACN] = 0.1 g L^-1^, [PMS] = 75 µM, [Pollutants] = 10 µM, initial pH = 7.0 ± 0.2.


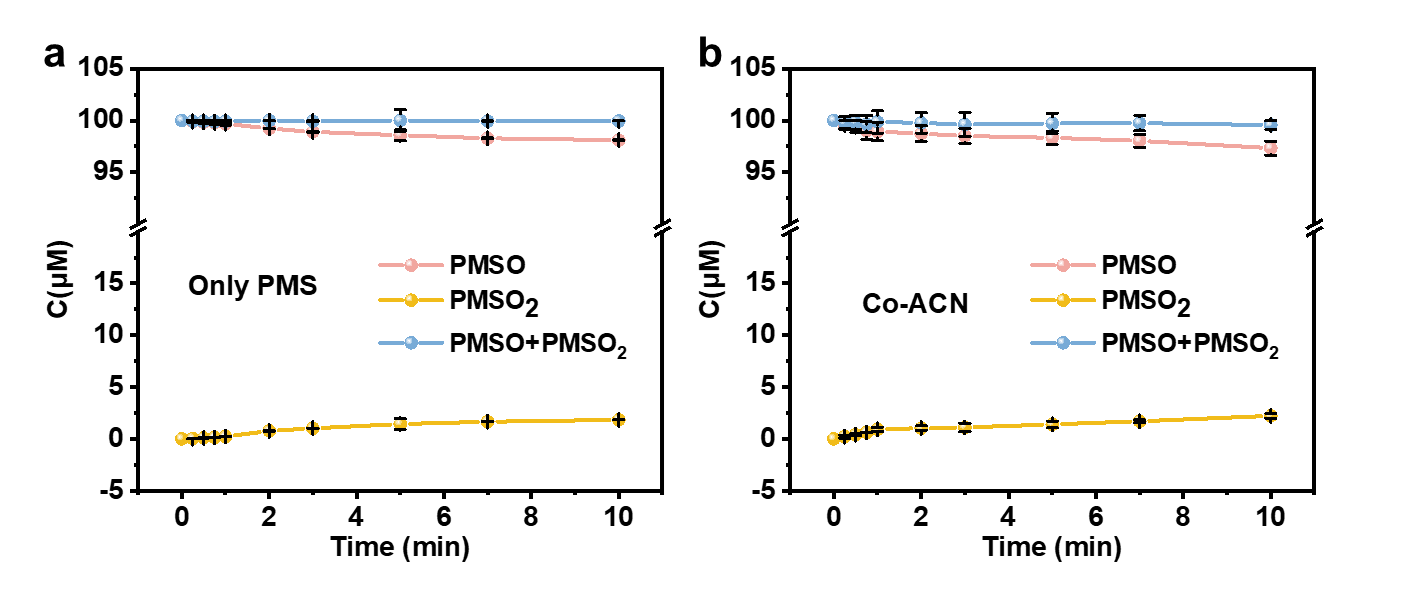


**Figure S22.** The consumption rate of PMSO and the generation rate of PMSO_2_ in the (a) PMS system and (b) Co-ACN/PMS system. [PMSO] = 0.1 mM, [Co-ACN] = 0.1 g L^-1^, [PMS] = 75 µM, initial pH = 7.0 ± 0.2.

As shown in Figure S22, approximately 2% of PMSO was consumed in the only PMS alone system, while the consumption of PMSO in the Co-ACN/PMS system was similar to that in the PMS alone system. Since PMS can also react with PMSO to form PMSO_2_, albeit at a slower oxidation rate, these results suggest that the Co-ACN catalyst did not participate in the consumption of PMSO. Consequently, high-valent cobalt-oxo oxidation was not involved in the Co-ACN/PMS system.

**^^**

**Figure S23.** The premixing experiment in Co-ACN/PMS system. [Co-ACN] = 0.1 g L^-1^, [PMS] = 75 µM, [Pollutants] = 10 µM, initial pH = 7.0 ± 0.2.

In the Co-ACN/PMS system, prolonged premixing time significantly reduces PZF degradation, attributed to the continuous consumption of ^1^O_2_. These results confirm that the ^1^O_2_ is the dominant reactive oxygen species in PZF degradation, excluding the impact of ETP.

**
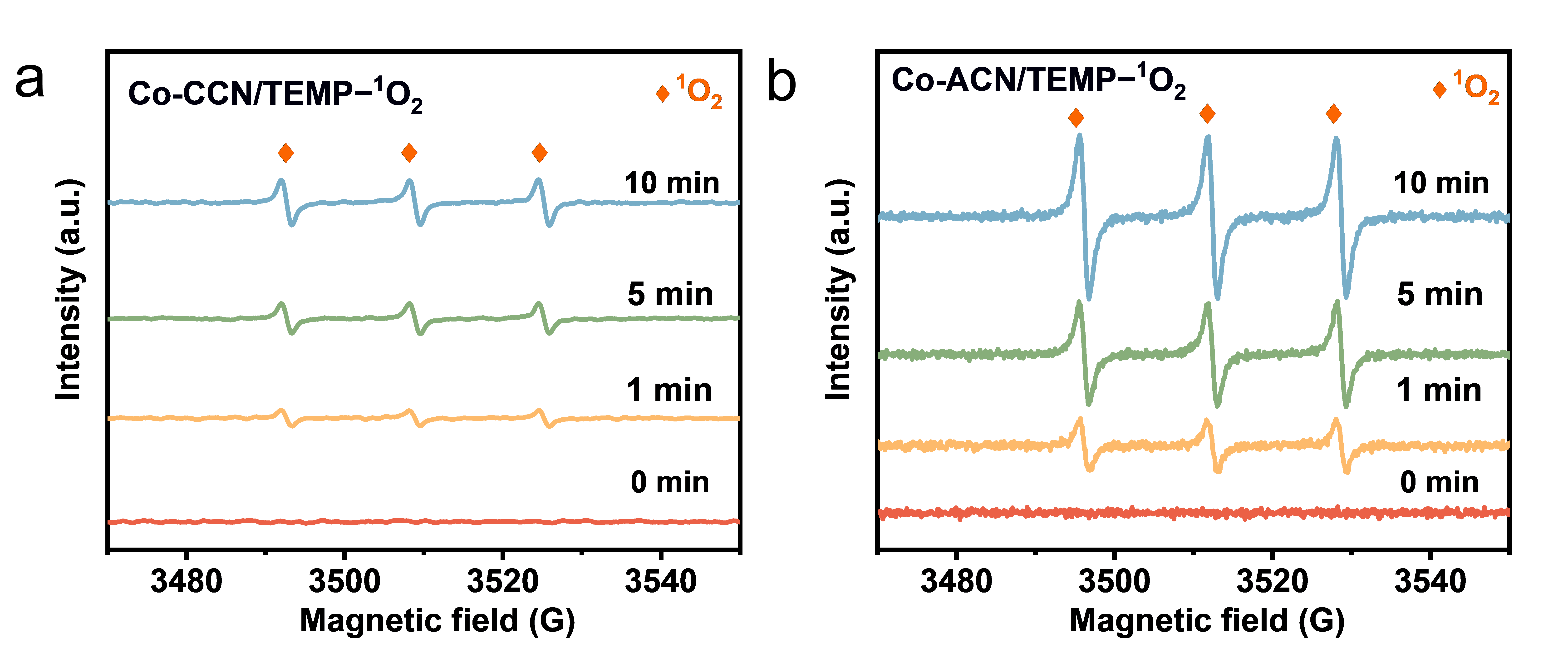
**

**Figure S24.** EPR spectra of TEMP-^1^O_2_ (a) Co-CCN, (b) Co-ACN.


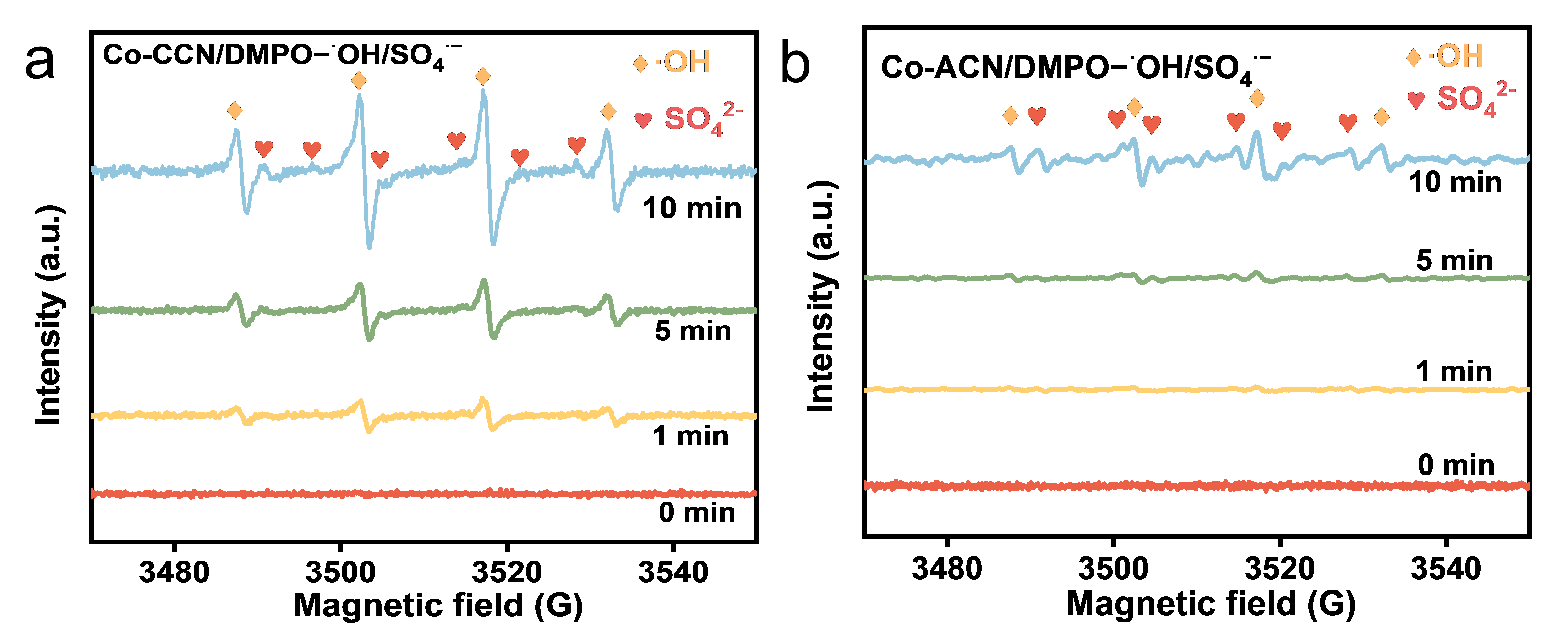


**Figure S25.** EPR spectra of DMPO-^•^OH/SO_4_^•−^ (a) Co-CCN, (b) Co-ACN.


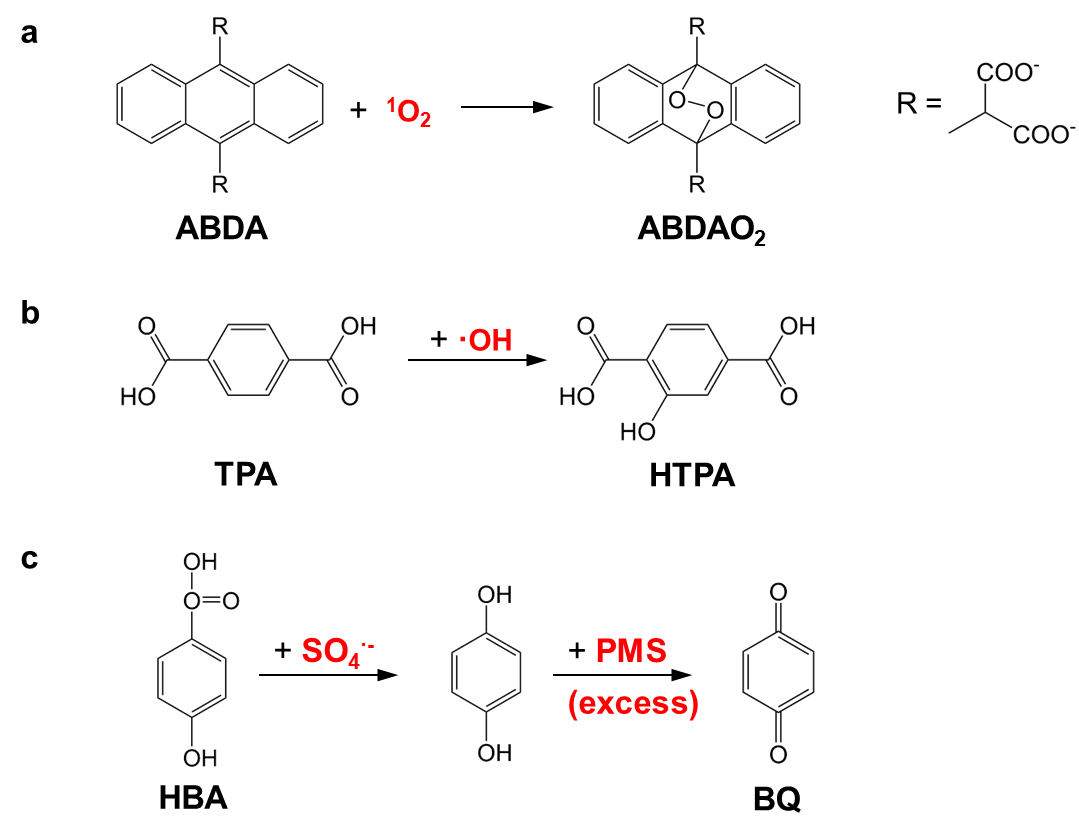


**Figure S26.** Chemical equation of (a) 9,10-anthracene-diyl-bis(methylene) dimalonic acid and ^1^O_2_, (b) terephthalic acid and ^•^OH, and (c) p-Hydroxybenzonic acid and SO_4_^•−^_._

*Quantitative ^1^O_2_:* Solution test with UV-Vis absorbance measurement for ^1^O_2_ detection. UV-Vis spectra were recorded using a Jasco V-750 spectrophotometer. The chemical probe 9,10-anthracene-diyl-bis(methylene) dimalonic acid (ABDA) was employed for the detection of ^1^O_2_. Initially, an ABDA indicator solution with a concentration of 1 mg/mL was prepared. Since the catalyst itself exhibits absorption characteristics, 10 mg of Co-ACN or Co-CCN was fully dispersed in 95 mL of deionized water before measurement. The baseline was first recorded, and then 5 mL ABDA was added to the reaction system. The UV-Vis spectra were measured at various reaction times, and the concentration of ^1^O_2_ was calculated according to the standard curve.

*Quantitative SO_4_^•−^:* The amount of SO_4_^•−^ generated in the Co-ACN/PMS or Co-CCN/PMS system was detected using p-Hydroxybenzonic acid (HBA) as a chemical probe. Based on the reaction stoichiometry, 1 mol of HBA reacts with 1 mol of SO_4_^•−^ to form hydroquinone which is immediately transformed to a stable by product, namely 1,4-Benzoquinone (BQ) by the excess PMS. Ultimately, the BQ in the system is detected by HPLC and the concentration of SO_4_^•−^ can be determined after conversion.

*Quantitative ^•^OH:* The concentration of ^•^OH generated during the AOP process is quantified by Liquid photoluminescence (PL) Spectra. Terephthalic acid (TPA) can capture ^•^OH to form 2-hydroxyterephthalic acid (HTPA) with strong fluorescent signal. The quantification system was consistent with the degradation experiments except that benzoic acid was used instead of pollutants ([catalyst] = 0.1 L^−1^; [Temp] = 25 °C; initial pH = 7.0 ± 0.2). After adsorption for 30 min to reach equilibrium, PMS was added to the reaction system (the concentration of PMS in the system was 0.075 mM). The solution is collected at intervals, filtered, and then measured by PL emission spectroscopy at excitation wavelength of 330 nm.


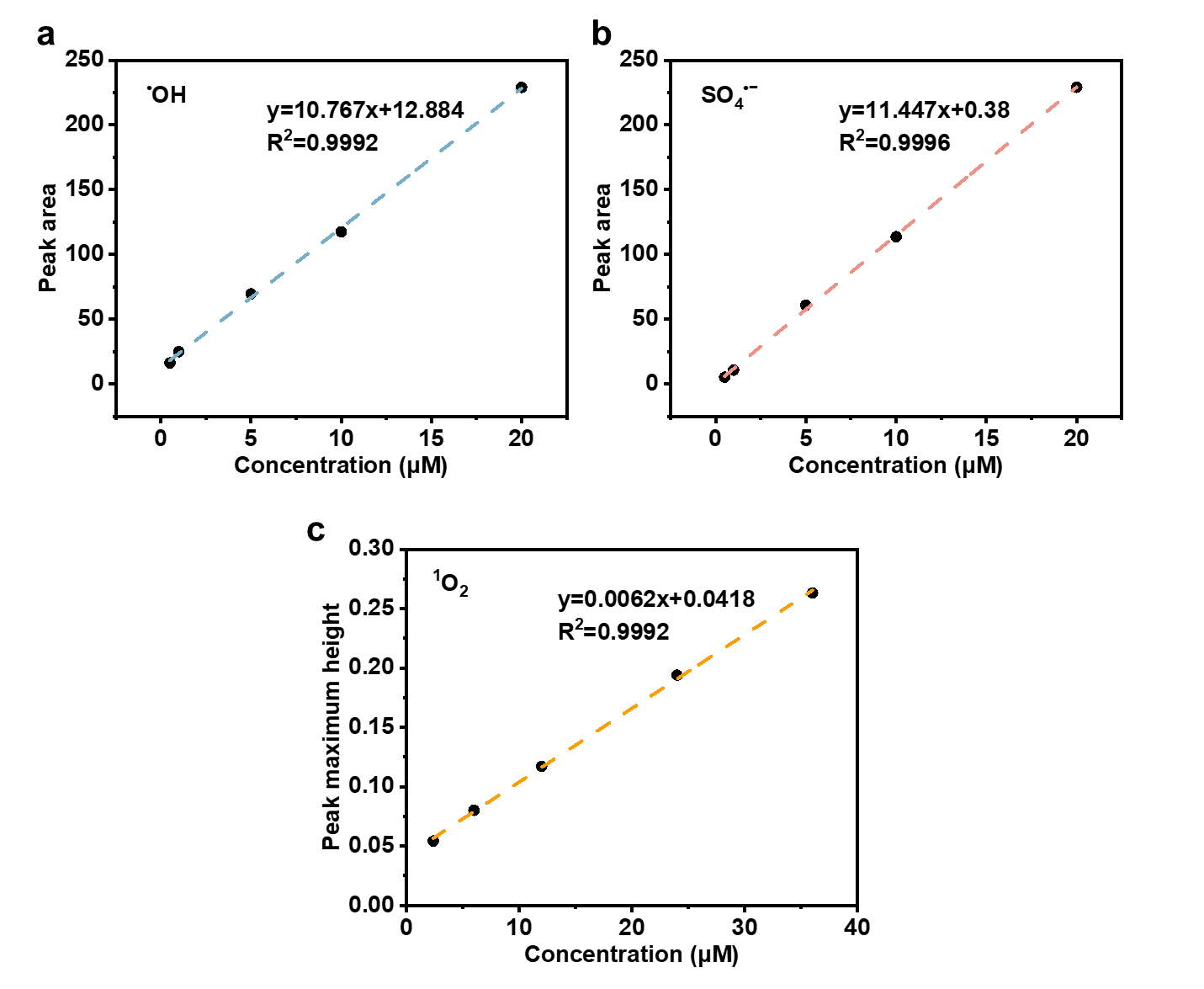


**Figure S27.** Standard curve in the quantitative analysis of reactive oxygen species. (a) ^•^OH, (b) SO_4_^•−^_._and (c) ^1^O_2_.

**Figure S28.** Linear Sweep Voltammetry (LSV) analysis was performed on the Co-CCN. [Co-ACN] = 0.1 g L^-1^, [PMS] = 75 µM.

**Reference**

[1] a) G. Kresse, J. Furthmüller, *Comput. Mater. Sci* **1996**, *6*, 15-50; b) G. Kresse, J. Furthmüller, *Phys. Rev. B* **1996**, *54*, 11169-11186.

[2] a) J. P. Perdew, K. Burke, M. Ernzerhof, *Phys. Rev. Lett.* **1996**, *77*, 3865-3868; b) J. P. Perdew, M. Ernzerhof, K. Burke, *J. Chem. Phys* **1996**, *105*, 9982-9985.

[3] a) S. Grimme, J. Antony, S. Ehrlich, H. Krieg, *J. Chem. Phys.* **2010**, *132*, 154104; b) S. Grimme, S. Ehrlich, L. Goerigk, *J. Comput. Chem.* **2011**, *32*, 1456-1465.

[4] P. E. Blöchl, *Phys. Rev. B* **1994**, *50*, 17953-17979.

[5] A. Hjorth Larsen, J. Jorgen Mortensen, J. Blomqvist, I. E. Castelli, R. Christensen, M. Dulak, J. Friis, M. N. Groves, B. Hammer, C. Hargus, E. D. Hermes, P. C. Jennings, P. Bjerre Jensen, J. Kermode, J. R. Kitchin, E. Leonhard Kolsbjerg, J. Kubal, K. Kaasbjerg, S. Lysgaard, J. Bergmann Maronsson, T. Maxson, T. Olsen, L. Pastewka, A. Peterson, C. Rostgaard, J. Schiotz, O. Schutt, M. Strange, K. S. Thygesen, T. Vegge, L. Vilhelmsen, M. Walter, Z. Zeng, K. W. Jacobsen, *J Phys Condens Matter* **2017**, *29*, 273002.

[6] a) G. Henkelman, A. Arnaldsson, H. Jónsson, *Comput. Mater. Sci.* **2006**, *36*, 354-360; b) E. Sanville, S. D. Kenny, R. Smith, G. Henkelman, *J. Comput. Chem.* **2007**, *28*, 899-908.

[7] V. Wang, N. Xu, J.-C. Liu, G. Tang, W.-T. Geng, *Comput. Phys. Commun.* **2021**, *267*, 108033.

[8] K. Momma, F. Izumi, *J. Appl. Crystallogr.* **2008**, *41*, 653-658.

[9] B. Huang, X. Ren, J. Zhao, Z. Wu, X. Wang, X. Song, X. Li, B. Liu, Z. Xiong, B. Lai, *Environ. Sci. Technol.* **2023**, *57*, 14071-14081.

[10] S. Wang, J. Wang, *Chem. Eng. J.* **2020**, *385,* 123933.

[11] Y. Bao, W.-D. Oh, T.-T. Lim, R. Wang, R. D. Webster, X. Hu, *Water Res.* **2019**, *151*, 64-74.

[12] X. Liang, D. Wang, Z. Zhao, T. Li, Y. Gao, C. Hu, *Adv. Funct. Mater.* **2022**, *32,* 2203001.

[13] X. Zhou, M.-K. Ke, G.-X. Huang, C. Chen, W. Chen, K. Liang, Y. Qu, J. Yang, Y. Wang, F. Li, H.-Q. Yu, Y. Wu, *Proc. Natl. Acad. Sci. U.S.A.* **2022**, *119(8),* e2119492119.

[14] A. Shahzad, J. Ali, J. Ifthikar, G. G. Aregay, J. Zhu, Z. Chen, Z. Chen, *J. Hazard. Mater.* **2020**, *392,* 122316.

[15] X. Fu, Q. Zeng, Y. Gao, L. Song, Y. Wen, T. Cai, Q. Zhang, C. Hu, Q. Zeng, *ACS ES&T Eng.* **2024**, *4*, 903-914.

[16] F. Wang, Y. Gao, H. Fu, S.-S. Liu, Y. Wei, P. Wang, C. Zhao, J.-F. Wang, C.-C. Wang, *Appl. Catal. B* **2023**, *339*, 123178.

[17] Z. Wang, E. Almatrafi, H. Wang, H. Qin, W. Wang, L. Du, S. Chen, G. Zeng, P. Xu, *Angew. Chem. Int. Ed.* **2022**, *61*, e202202338.

[18] S. Liu, J. Du, H. Wang, W. Jia, Y. Wu, P. Qi, S. Zhan, Q. Wu, J. Ma, N. Ren, W.-Q. Guo, *Water Res.* **2024**, *254*, 121417.

[19] G. Bi, R. Ding, J. Song, M. Luo, H. Zhang, M. Liu, D. Huang, Y. Mu, *Angew. Chem. Int. Ed.* **2024**, *63*, e202401551.

[20] Y. Kang, Y. Yang, L.-C. Yin, X. Kang, G. Liu, H.-M. Cheng, *Adv. Mater.* **2015**, *27*, 4572-4577.

[21] E. J. Son, Y. W. Lee, J. W. Ko, C. B. Park, *ACS Sustainable Chem. Eng.* **2019**, *7*, 2545-2552.

[22] X. Xiao, Y. Gao, L. Zhang, J. Zhang, Q. Zhang, Q. Li, H. Bao, J. Zhou, S. Miao, N. Chen, J. Wang, B. Jiang, C. Tian, H. Fu, *Adv. Mater.* **2020**, *32*, 2003082.
